# C-type lectin-mediated microbial homeostasis is critical for *Helicoverpa armigera* larval growth and development

**DOI:** 10.1371/journal.ppat.1008901

**Published:** 2020-09-30

**Authors:** Wenwen Wang, Guijie Wang, Xiaorong Zhuo, Yu Liu, Lin Tang, Xusheng Liu, Jialin Wang

**Affiliations:** Hubei Key Laboratory of Genetic Regulation and Integrative Biology, School of Life Sciences, Central China Normal University, Wuhan, China; Pennsylvania State University, UNITED STATES

## Abstract

The immune system of a host functions critically in shaping the composition of the microbiota, and some microbes are involved in regulating host endocrine system and development. However, whether the immune system acts on endocrine and development by shaping the composition of the microbiota remains unclear, and few molecular players or microbes involved in this process have been identified. In the current study, we found that RNA interference of a C-type lectin (HaCTL3) in the cotton bollworm *Helicoverpa armigera* suppresses ecdysone and juvenile hormone signaling, thus reducing larval body size and delaying pupation. Depletion of HaCTL3 also results in an increased abundance of *Enterocuccus mundtii* in the hemolymph, which may escape from the gut. Furthermore, HaCTL3 and its controlled antimicrobial peptides (attacin, lebocin, and gloverin) are involved in the clearance of *E*. *mundtii* from the hemolymph via phagocytosis or direct bactericidal activity. Injection of *E*. *mundtii* into larval hemocoel mimics HaCTL3-depleted phenotypes and suppresses ecdysone and juvenile hormone signaling. Taken together, we conclude that HaCTL3 maintains normal larval growth and development of *H*. *armigera* via suppressing the abundance of *E*. *mundtii* in the hemolymph. Our results provide the first evidence of an immune system acting on an endocrine system to modulate development via shaping the composition of microbiota in insect hemolymph. Thus, this study will deepen our understanding of the interaction between immunity and development.

## Author summary

Considering that a large number of hemocytes and multiple soluble effectors are present in insect hemolymph, it is conventionally believed that healthy insect hemolymph is a hostile environment for bacteria and is, therefore, sterile. However, increasing evidences disprove this opinion, although the interactive mechanism between hemolymph microbiota and insect host, as well as the function of hemolymph microbiota, remain unclear. C-type lectin (CTL), as pattern recognition receptor (PRR), plays important roles in defending against various pathogens. Here we found that various bacteria colonized the hemolymph of the cotton bollworm *Helicoverpa armigera*. We first reported that an *H*. *armigera* CTL (HaCTL3) was involved in modulating larval growth and development. Further study indicated that HaCTL3-mediated homeostasis of *Enterocuccus mundtii* in the hemolymph is critical for normal larval growth and development. Our study demonstrated that this PRR modulated insect development through shaping hemolymph microbiota, which may represent a novel mechanism of immune system regulation during insect development.

## Introduction

The immune capacity of vertebrates and invertebrates varies at different developmental stages of life, known as age-dependent immunity. In mammals, immune cell migration and pattern recognition receptor (PRR) signaling response to pathogens are impaired in aged individuals [1]. In *Drosophila melanogaster* and *Aedes aegypti*, the number of hemocytes decreases with age, resulting in increased susceptibility to pathogen infection [2, 3]. Many studies suggest that immune capacity varies at different developmental stages largely due to the varied levels of endocrine regulators such as hormones. The steroid hormone glucocorticoid exhibits anti-inflammatory and immunosuppressive properties in mammals [4, 5]. However, physiological levels of glucocorticoid are reported to promote inflammatory and immune responses [6, 7]. In insects, the steroid hormone 20-hydroxyecdysone (20E) positively regulates innate immunity [8–10], whereas juvenile hormone (JH) serves as an immune-suppressor [11].

Despite the availability of studies regarding endocrine regulation of immunity, information regarding the impact of immunity on the endocrine system and development is quite limited. Hypothalamic IκB kinase-β and nuclear factor κB regulate mammalian lifespan extension by inhibiting gonadotropin-releasing hormone, representing immune-neuroendocrine integration [12]. In addition, all metazoan guts harbor various commensal bacteria, some of which has the ability to modulate host endocrine and development. For example, *Acetobacter pomorum* in the gut regulates the developmental rate and body size of its host *Drosophila* via insulin signaling [13]. Another gut commensal bacteria *Lactobacillus plantarum* enhances hormonal growth signaling in *Drosophila* and promotes systemic growth [14]. In some insects, symbiotic bacteria are found not only in the intestines but also in the hemolymph [15–17]. The insect host bacterial community in the hemolymph may be influenced by the diet [18]. When a fall armyworm feeds on resistant maize, its gut microbes penetrate the intestinal barriers and invade the body cavity [19]. Some hemolymph bacteria are translocated from the gut through *Bacillus thuringiensis* toxin-induced epithelial lesions, changing the roles from resident symbionts in the gut to pathogens in the hemolymph [20–22]. Given that some microbes play crucial roles in modulating host development, molecular players involved in shaping the host bacterial community have the potential to regulate host development.

PRRs recognize conserved microbial components known as pathogen-associated molecular patterns (PAMPs) such as lipopolysaccharide (LPS) and peptidoglycan (PGN). There are several types of PRRs, including β-1,3-glucan recognition proteins (β-1,3-GRPs), scavenger receptors (SRs), peptidoglycan recognition proteins (PGRPs), and C-type lectins (CTLs) [23]. Various immune responses such as phagocytosis, encapsulation, prophenoloxidase activation, and synthesis of antimicrobial peptides (AMPs) are initiated following recognition of PAMPs by PRRs [24, 25]. PRRs are also involved in maintaining microbiome homeostasis in the gut and hemolymph. In the mosquito, depletion of PGRP-LB increases the amount of gut microbiota, suggesting its role in controlling gut microbial homeostasis [26]. Mosquito PGRP-LC stimulates immune-deficiency pathways thereby limiting the gut microbiota [27]. The gut microbiome activates the expression of mosquito CTL, which helps the microbiome to evade the bactericidal capacity of AMPs and facilitates colonization [28]. A shrimp CTL functions in restraining the hemolymph microbiota by maintaining the expression of AMPs [29].

CTLs in invertebrates are more abundant and diverse than those in vertebrates, probably reflecting a heavier burden on pathogen recognition [30, 31]. A total of 26 CTLs have been identified in the cotton bollworm, *Helicoverpa armigera*, based on transcriptomic analyses of immune-related genes [32, 33]. We have previously shown that *H*. *armigera* CTL3 (HaCTL3, GenBank accession No. AFI47448) can agglutinate various bacteria and facilitate hemocytic encapsulation [32, 34]. Here, we demonstrated that depletion of HaCTL3 using RNA interference (RNAi) reduced larval body size, delayed pupation, and increased hemolymph load of gram-positive *Enterocuccus mundtii*. HaCTL3 and its controlled AMPs participated in clearance of *E*. *mundtii* by phagocytosis and bactericidal activity. We confirmed that injection of *E*. *mundtii* into the hemocoel mimics HaCTL3-depleted phenotypes and suppresses hormonal signaling. Our results therefore reveal a novel mechanism by which the immune system acts on the endocrine system to regulate insect growth and development.

## Results

### HaCTL3 modulates larval growth and development

#### Depletion of HaCTL3 reduces larval body size and delays pupation time

**Fig 1:**
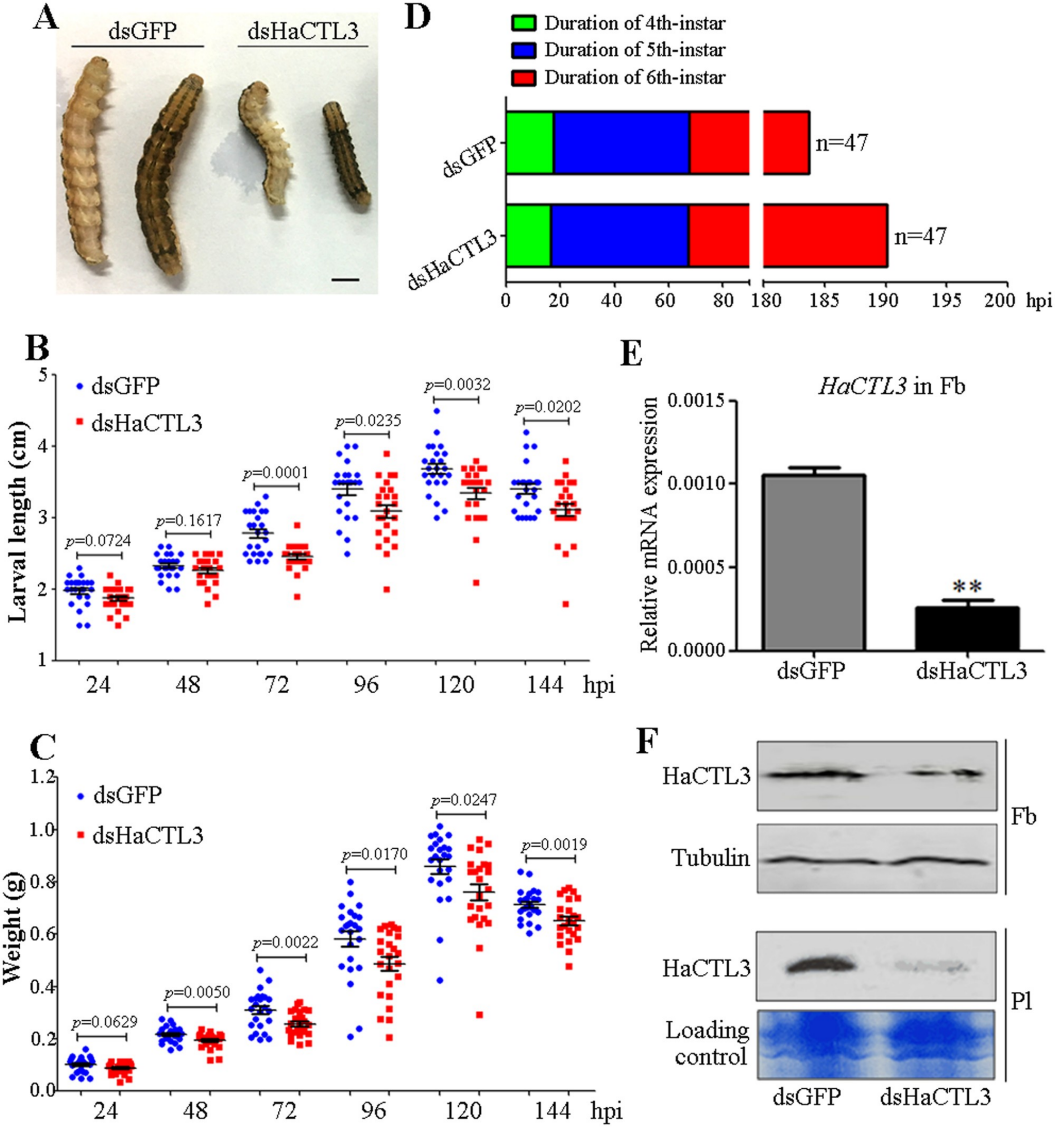
Reduced body size and delayed pupation in HaCTL3-depleted larvae. (A) The size of representative dsHaCTL3- or dsGFP-injected larva. Larvae were photographed at 72 h post-dsRNA injection (hpi). Bar = 0.5 cm. (B, C) Larval body length (B) and weight (C) were reduced in HaCTL3-depleted larvae. Larval body length and weight were measured at 24, 48, 72, 96, 120, and 144 hpi. The *p* value was calculated by the Student’s *t* test for paired samples, and a *p* value of < 0.05 was considered statistically significant. (D) Delayed pupation time in HaCTL3-depleted larvae. The duration of fourth-, fifth-, and sixth-instar was measured based on 47 dsHaCTL3- or dsGFP-injected larvae. (E) RT-qPCR analysis showing the depletion efficiency of *HaCTL3* transcripts in the fat body. The bar represents mean ± SEM from three biological replicates. **0.001 < *p* < 0.01 (Student’s *t*-test). (F) Western blot confirming the reduced level of HaCTL3 proteins in the fat body (Fb) and plasma (Pl).

To determine the function of HaCTL3, we injected dsRNA targeting *HaCTL3* (dsHaCTL3) or *green fluorescence protein* (dsGFP, control) into the hemocoel of fourth-instar *H*. *armigera* larvae. Surprisingly, we observed a visibly reduced body size after HaCTL3 depletion (Fig 1A). Larvae showed significantly smaller length at 72, 96, 120, and 144 h post-dsHaCTL3 injection compared with that of the control groups (Fig 1B). Larvae also exhibited significantly reduced weight at 48, 72, 96, 120, and 144 h post-dsHaCTL3 injection (Fig 1C). The duration of fourth- or fifth-instar between the dsHaCTL3-injected group and the control group exhibited no obvious differences; however, the HaCTL3-depleted group showed delayed pupation for an average of approximately 6 h (Fig 1D). Because HaCTL3 is mainly expressed in the fat body and is subsequently secreted into the hemolymph [32, 34], we confirmed the successful knockdown of HaCTL3 transcripts and proteins in fat body (Fig 1E and 1F). Efficient knockdown of HaCTL3 in plasma was also confirmed by western blot (Fig 1F).

To exclude the possible off-target effect of RNAi, we synthesized another fragment of dsRNA against HaCTL3 (dsHaCTL3-2) that does not overlap with dsHaCTL3. The expression level of HaCTL3 in the fat body was successfully silenced by injection of dsHaCTL3-2 (S1A and S1B Fig). The larval length and weight were significantly reduced (S1C and S1D Fig), and the pupation time was delayed (S1E Fig). Taken together, we concluded that HaCTL3 depletion suppresses larval growth and delays pupation.

#### Depletion of HaCTL3 suppresses 20E and JH signaling

Because growth duration and growth rate are mainly controlled by ecdysone and JH [35, 36], we investigated the titres of these two hormones. Ecdysone titres and the genes involved in 20E signaling were tested at the wandering stage, whereas JH titres and the genes involved in JH signaling were determined at the feeding stage of sixth-instar larvae.

**Fig 2:**
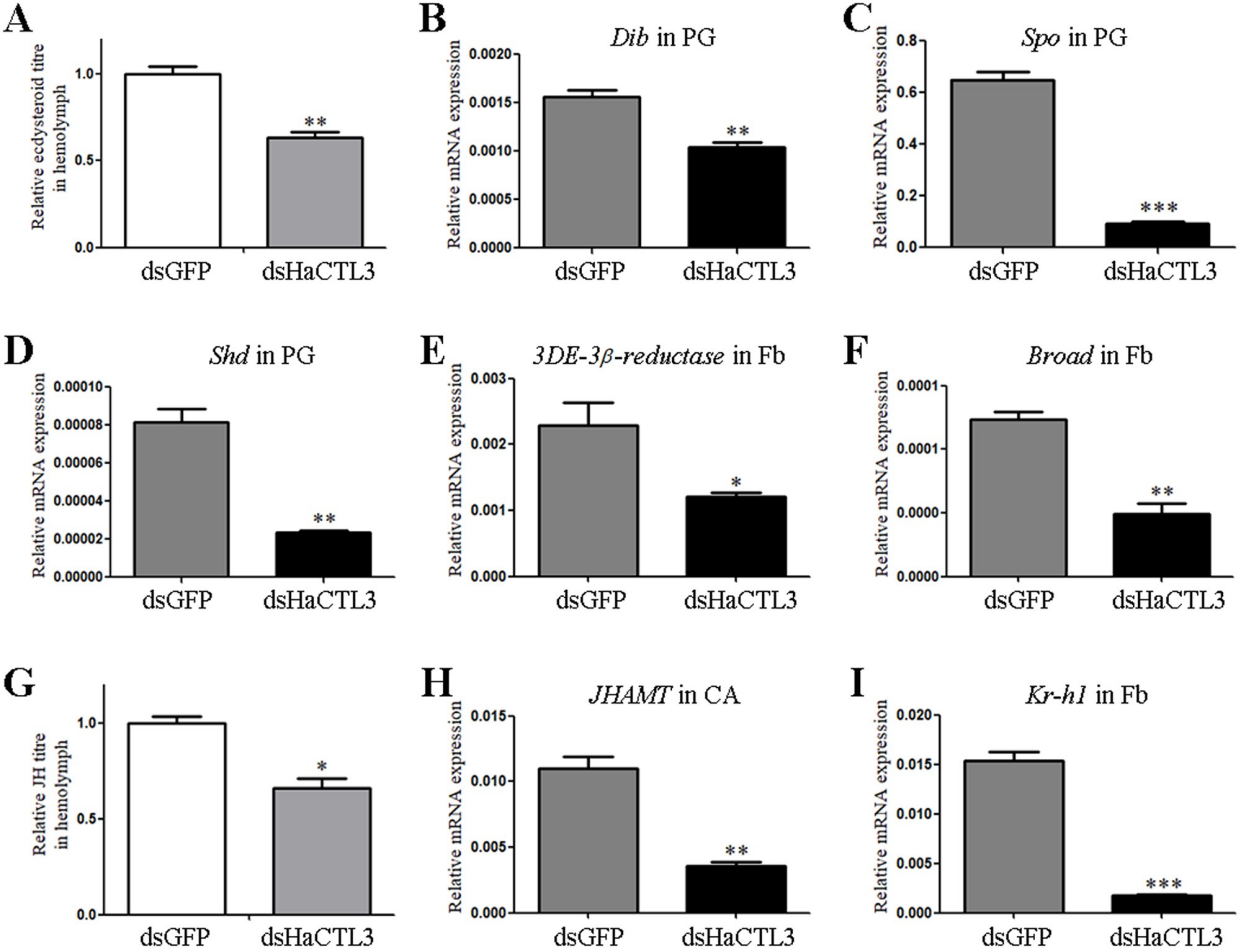
Suppression in 20E and JH signaling in HaCTL3-depleted larvae. (A, G) Measurement of relative ecdysone titres (A) and JH titres (G) in the hemolymph of dsHaCTL3- or dsGFP-treated larvae. (B–F, H, I) Reduction in transcripts of *Dib* (B), *Spo* (C), and *Shd* (D) in PG, *3DE-3β-reductase* (E), *Broad* (F), and *Kr-h1* (I) in Fb, and *JHAMT* (H) in CA of dsHaCTL3-treated larvae. PG, prothoracic gland; Fb, fat body; CA, corpora allata. The bar represents mean ± SEM of three biological replicates. *0.01 < *p* < 0.05, **0.001 < *p* < 0.01, ***p < 0.001 (Student’s *t*-test).

Ecdysone titres in the hemolymph collected from HaCTL3-depleted larvae were significantly lower than those from the control group (Fig 2A). We examined the expression of genes involved in ecdysteroid biosynthesis in the prothoracic gland (PG) such as *Disembodied* (*Dib*), *Spook* (*Spo*), and *Shade* (*Shd*). As shown in Fig 2B–2D, the expression of *Dib*, *Spo*, and *Shd* was downregulated in HaCTL3-depleted larvae. Because 3-dehydroecdysone 3β-reductase (3DE-3β-reductase) plays a crucial role in reducing 3DE to ecdysone in the hemolymph [37], we tested the expression of this gene and found that *3DE-3β-reductase* was significantly decreased in the HaCTL3-depleted fat body (Fig 2E). It was reported that the ecdysone-response gene *Broad* dictates pupal commitment [38, 39]. The abundance of *Broad* transcripts was significantly decreased in HaCTL3-depleted larvae (Fig 2F), which reflected suppressed 20E signaling and was consistent with the delayed pupation time.

JH titres in HaCTL3-depleted hemolymph were significantly lower than those in the control group (Fig 2G). JH acid methyltransferase (JHAMT) is a key enzyme that converts JH acids into active JH at the final step of JH biosynthesis [40, 41]. The expression of *JHAMT* was decreased in the corpora allata (CA) of HaCTL3-depleted larvae (Fig 2H). The expression of JH-early inducible gene *krüppel homolog 1* (*Kr-h1*) was also downregulated (Fig 2I), further reflecting suppressed JH signaling in HaCTL3-depleted larvae.

#### Administration of 20E and/or JH rescues HaCTL3-depleted phenotypes

Since HaCTL3 depletion reduced larval body size, delayed pupation, and suppressed 20E and JH titres, we wonder whether administration of 20E and/or JH rescues HaCTL3-depleted phenotypes. Larvae pretreated with dsHaCTL3 exhibited no significant differences in larval length and weight at 0 h post-injection of JH or solvent (control). However, the length and weight of HaCTL3-depleted larvae were significantly increased at 48 h post-JH injection compared with the control group (S2A and S2B Fig). Further, the duration of sixth-instar between 20E-injected group and the control group exhibited no obvious differences. However, administration of JH and JH plus 20E accelerated pupation of HaCTL3-depleted larvae for an average of approximately 4 and 5 h, respectively (S2C Fig). Therefore, we concluded that the reduced body size in HaCTL3-depleted larvae might be largely due to the suppressed JH titers, and the delayed pupation might be attributable to the suppressed 20E and JH titres.

### HaCTL3 maintains low level of *E*. *mundtii* in hemolymph

#### Depletion of HaCTL3 results in a significant increase of Enterococcaceae abundance in larval hemolymph

CTL as a PRR may function in shaping the composition of the bacteria, and some of bacteria are involved in modulating host’s development [13, 14, 29]. Hence, we associated the abnormal phenotypes caused by HaCTL3 depletion with bacteria. To test whether *H*. *armigera* hemolymph was colonized by bacteria and to compare the composition and diversity of microbiota, samples of sixth-instar larvae at 24 h post-ecdysis (PE; feeding stage) and 96 h PE (wandering stage) were subjected to deep-sequencing analysis. The number of operational taxonomic units (OTUs) in the wandering stage was much less than that in the feeding stage, with 11 unique OTUs in the former and 1381 unique OTUs in the latter (S3 Fig). This suggests that hemolymph has much higher species diversity at the feeding stage than at the wandering stage. There were 84 OTUs shared between the feeding stage and the wandering stage (S3 Fig). The 1476 total OTUs were annotated into 29 phyla, 61 classes, 129 orders, 242 families, 531 genera, and 846 species (S1 Table).

To test whether HaCTL3 was involved in shaping microbiota in the hemolymph, we compared the composition and diversity of hemolymph microbiota between HaCTL3-depleted samples and the controls at 144 h post-dsRNA injection and found the 31 shared OTUs. There were 155 unique OTUs identified in the HaCTL3-depleted group, an amount much more abundant than that (12 unique OTUs) in the control (dsGFP) group (S3 Fig). This suggests that HaCTL3 plays critical roles in eliminating hemolymph microbiota overall. The 198 total OTUs represented 12 phyla, 19 classes, 41 orders, 66 families, 121 genera, and 164 species (S2 Table).

**Fig 3:**
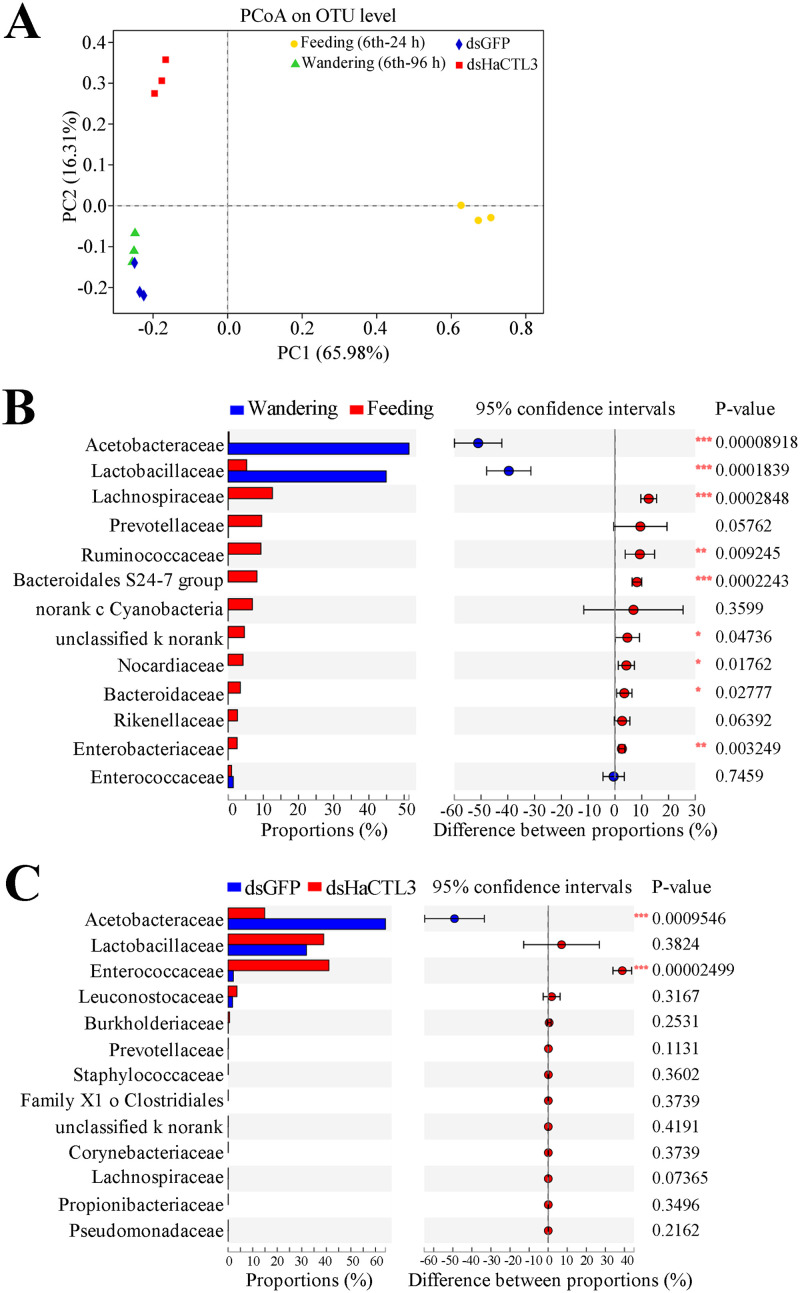
HaCTL3 contributes to maintaining a high abundance of Acetobacteraceae and a low abundance of Enterococcaceae in larval hemolymph. (A) Principal coordinates analysis (PCoA) of microbial communities from hemolymph based on OTU abundance. Hemolymph was collected from sixth-instar larvae at 24 h PE (6th-24 h; Feeding) and 96 h PE (6th-96 h; Wandering), as well as from larvae of dsHaCTL3- or dsGFP-injected (as control) groups. (B) Relative abundance (%) of family between the feeding stage and the wandering stage. The abundance of Acetobacteraceae and Lactobacillaceae significantly increased when larvae transitioned from the feeding stage to the wandering stage. (C) Relative abundance (%) of family between HaCTL3-depleted and the control group. Depletion of HaCTL3 significantly increased Enterococcaceae abundance and decreased Acetobacteraceae abundance during the wandering stage. The most abundant 13 families are shown. Statistical analysis was performed by Student’s *t*-test with *0.01 < *p* < 0.05, **0.001 < *p* < 0.01, and ***p < 0.001.

Principal coordinates analysis (PCoA, Bray–Curtis) further revealed that the structure of hemolymph microbiota is different between the feeding and the wandering stages, as well as between the HaCTL3-depleted and the control (dsGFP) groups (Fig 3A). Hemolymph of dsGFP group collected at 144 h post-dsRNA injection corresponds to the wandering stage, accounting for the similarity of the structure of hemolymph microbiota between the dsGFP group and samples in the wandering stage (Fig 3A).

Most microbes decreased in abundance after entering from the feeding stage to the wandering stage at the family level (Fig 3B; S1 Table). However, Acetobacteraceae and Lactobacillaceae markedly increased in abundance and resulted in almost exclusive colonization during the wandering stage (Fig 3B), suggesting that these bacteria may play important roles during the wandering stage.

Most microbes increased in abundance after HaCTL3 depletion during the wandering stage, with Enterococcaceae exhibiting the greatest increase and becoming the dominant bacterium (Fig 3C; S2 Table). HaCTL3 depletion largely decreased Acetobacteraceae abundance in hemolymph. There were no significant differences in the abundance of Lactobacillaceae between the HaCTL3-depleted group and the control group (Fig 3C).

#### HaCTL3 promotes hemocytic phagocytosis of *E*. *mundtii*

Because greater microbiota abundance and diversity in HaCTL3-depleted hemolymph was observed, we speculated that HaCTL3 is involved in bacterial clearance *in vivo*. To test this, we knocked down the expression of HaCTL3 followed by injection of gram-negative *Escherichia coli* or gram-positive *Staphylococcus aureus* into larval hemocoel. Hemolymph was collected at 1 h post-bacterial injection, and relative bacterial numbers were compared. The numbers of both *E*. *coli* and *S*. *aureus* colony-forming units (CFUs) in the hemolymph of HaCTL3-depleted larvae were higher than those of the control groups (S4A Fig), suggesting that HaCTL3 promotes bacterial clearance in *H*. *armigera*.

**Fig 4:**
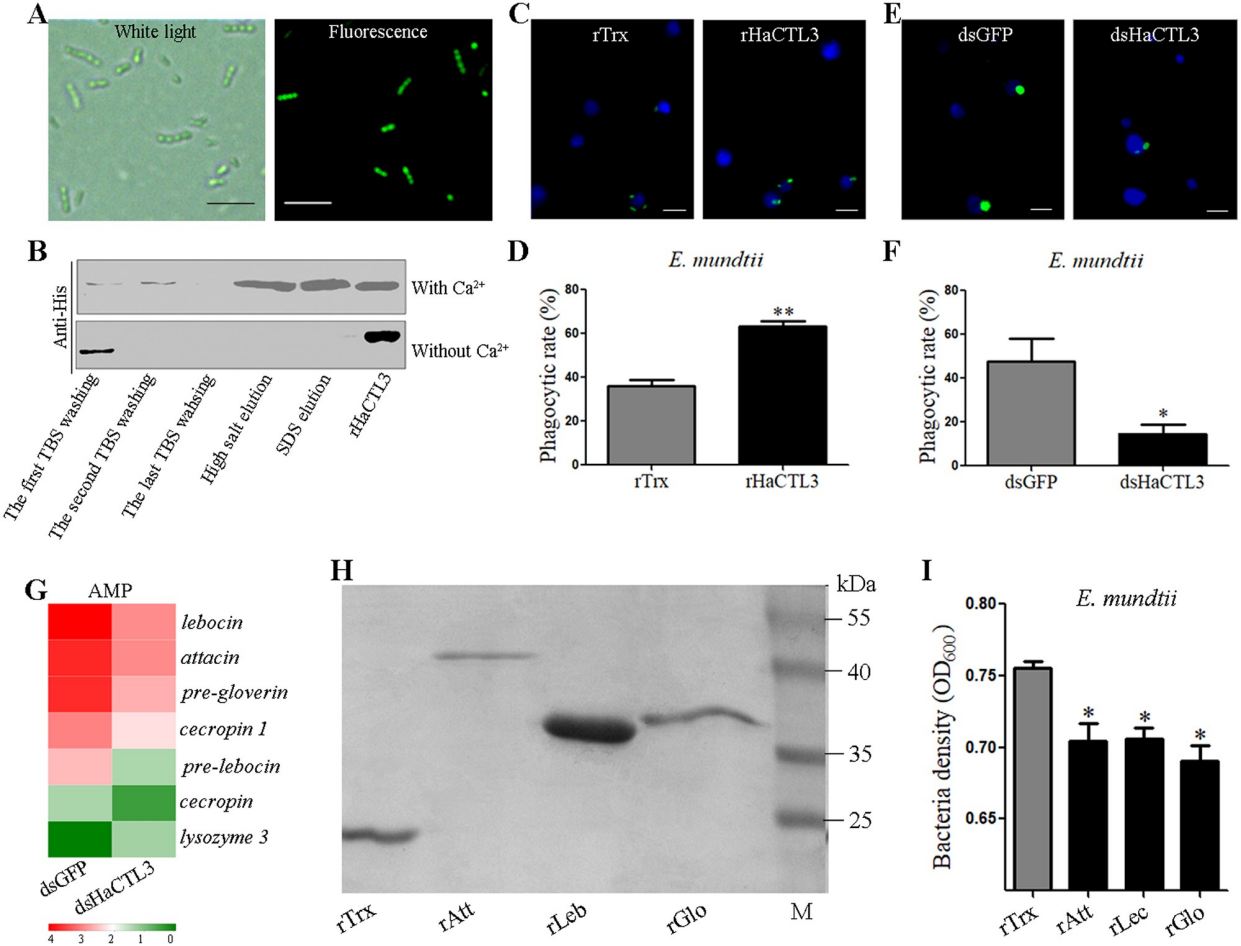
HaCTL3 and its controlled AMPs contribute to elimination and suppression of *E*. *mundtii* in hemolymph. (A) Morphological characterization of *E*. *mundtii*. Bacteria (FITC-stained) were photographed under white light and fluorescence microscopes. (B) Binding of rHaCTL3 to *E*. *mundtii* in the presence of Ca^2+^. *E*. *mundtii* were incubated with rHaCTL3 for 30 min. After washing with TBS, bacteria were pelleted and subjected to elution with high salt and SDS, respectively. The rHaCTL3 was loaded as control. (C) Representative images showing that rHaCTL3 promotes hemocytic phagocytosis of *E*. *mundtii*. FITC-labeled bacteria (green) were preincubated with rHaCTL3 or rTrx and then injected into the hemocoel of sixth-instar larvae at 24 h PE. (D) Statistical analysis of phagocytic rate toward rHaCTL3- or rTrx-coated *E*. *mundtii*. (E) Representative images indicating that HaCTL3 depletion suppresses hemocytic phagocytosis. Larvae at 96 h post-dsHaCTL3 or dsGFP treatment were injected with FITC-labeled *E*. *mundtii* (green). (F) Statistical analysis of phagocytic rate toward *E*. *mundtii* in dsHaCTL3- or dsGFP-injected larvae. DAPI was used to stain hemocyte nuclei (blue). Bars represent 10 μm. Statistical analysis was performed by Student’s *t*-test with *0.01 < *p* < 0.05 and **0.001 < *p* < 0.01. (G) Heatmap showing that most DEGs encoding AMPs were downregulated in HaCTL3-depleted fat body. (H) Purification of recombinant thioredoxin (rTrx), attacin (rAtt), lebocin (rLeb), and gloverin (rGlo). M, protein standard. (I) Recombinant AMPs exhibit direct bacteriostatic activity *in vitro*. rAtt, rLeb, rGlo, or rTrx (as control) were incubated with *E*. *mundtii* for 12 h, and bacterial growth was evaluated by measuring the absorbance at 600 nm. The *p* value was calculated by the Student’s *t* test for paired samples against controls (*0.01 < *p* < 0.05).

Because Enterococcaceae showed the greatest increase and became the dominant bacteria in HaCTL3-depleted hemolymph, we next sought to isolate Enterococcaceae by plating hemolymph onto Luria-Bertani (LB) agar plates. By selecting single colonies, amplifying DNA fragments with bacterial universal primers 27F and 1492R of the 16S ribosomal RNA (rRNA) gene, and gene sequencing, we eventually characterized the gram-positive *E*. *mundtii*, a member of Enterococcaceae. *E*. *mundtii* exists as a single bacterium with coccus morphology. The bacteria can also be organized in small chains of 2–5 cells (Fig 4A). To determine whether HaCTL3 directly binds to *E*. *mundtii*, bacteria were incubated with recombinant HaCTL3 (rHaCTL3), which was prepared previously [32], in the presence or absence of calcium (Ca^2+^). After washing and elution, samples were subjected to western blot analyses. HaCTL3 bound to *E*. *mundtii* in a Ca^2+^-dependent manner (Fig 4B). Binding of rHaCTL3 to gram-positive bacteria (*S*. *aureus*, *Microbacterium pumilum*, and *Cellulosimicrobium funkei*) and gram-negative bacteria (*E*. *coli*, *Vibrio anguillarum*, and *Ochrobactrum cytisi*) was also demonstrated in the presence of Ca^2+^ (S4B and S4C Fig), suggesting HaCTL3 has a wide bacterial binding activity. Further, PGN competitively inhibited the binding ability of rHaCTL3 to *S*. *aureus*, whereas trehalose, LPS, galactose, sucrose, maltose, mannose, and glucose did not (S4D Fig). Hence, we concluded that HaCTL3 directly binds various bacteria via certain PAMPs (such as PGN) as a PRR.

To test whether HaCTL3 is involved in phagocytosis, we tested the rate at which hemocytes phagocytosed *E*. *mundtii*, *E*. *coli*, and *S*. *aureus*. As shown in Fig 4C and 4D, and S4E Fig, the phagocytic rate toward rHaCTL3-coated *E*. *mundtii*, *E*. *coli*, or *S*. *aureus* was significantly higher than that of the control groups, and HaCTL3 depletion significantly decreased this phagocytic rate (Fig 4E and 4F, and S4F Fig). These results demonstrated that HaCTL3 promotes hemocytic phagocytosis of various bacteria (such as *E*. *mundtii*).

#### HaCTL3-controlled AMPs contribute to the suppression of *E*. *mundtii* in hemolymph

Because plasma and hemocytes constitute circulating hemolymph, we next wondered whether plasma contributes to bacterial clearance. To test this, the antibacterial activity of hemocyte-free plasma was determined by incubation with bacterial suspensions. HaCTL3 depletion significantly increased the number of bacterial colonies of both *E*. *coli* and *S*. *aureus* compared with those exposed to plasma with intact HaCTL3 (S4G Fig), suggesting that HaCTL3 enhances the antibacterial activity of the plasma.

To evaluate the effect of HaCTL3 on the expression of immune-related genes, we performed transcriptomic expression profiling of the fat body in response to HaCTL3 depletion. A total of 762 differentially expressed genes (DEGs) were identified in the HaCTL3-depleted fat body, including 453 upregulated and 309 downregulated DEGs (S5A Fig; S3 Table). The DEG encoding 3DE-3β-reductase was largely downregulated in the HaCTL3-depleted fat body (S3 Table), supporting the quantitative real-time reverse-transcriptase PCR (RT-qPCR) results (Fig 2E). Gene ontology (GO) analysis was conducted for functional categorization of these DEGs (S5B Fig; S4 Table). Within biological processes, the DEGs involved in the metabolic process (80, 29.63%) constituted the largest group, followed by the cellular process (56, 20.74%) and single-organism process (49, 18.15%). Most cellular components were grouped into cell (46, 21.70%) and cell part (46, 21.70%), whereas most molecular function groups were involved in catalytic activity (65, 47.79%) and binding (59, 43.38%). Interestingly, GO analysis indicated that DEGs involved in the immune system process were all downregulated in HaCTL3-depleted samples (S5B Fig).

To the best of our knowledge, of the 762 DEGs, there were 29 involved in immunity, with only 6 upregulated and 23 downregulated in the dsHaCTL3- *vs*. dsGFP-injected samples (S5 Table). Depletion of *HaCTL3* was confirmed, and seven other identified PRR genes (*β-1*,*3-GRP 2a*, *β-1*,*3-GRP 3*, *PGRP A*, *β-1*,*3-GRP 1*, *SR-C-like*, *CTL4*, and *PGRP C*) were downregulated (S5C Fig). Seven melanization-related DEGs (*serine protease inhibitor SPI dipetalogastin*, *SPI 28*, *SPI 3/4*; *hemolymph proteinases HP8*, *HP18*, *HP19*, *HP21 precursor*) were identified (S5D Fig). Seven immune-related DEGs (*mucin-2*; *mucin-2-like*; *Hdd1*; *GBPB*, *growth blocking peptide binding protein*; *TIP-like*, *toll-interacting protein-like*; *Spz1A*; *ECSIT*, *evolutionarily conserved signaling intermediate in Toll pathway*) were also identified (S5E Fig). All AMP genes (*lebocin*, *attacin*, *pre-gloverin*, *cecropin 1*, *pre-lebocin*, and *cecropin*) were downregulated with the exception of *lysozyme 3*, which was upregulated in HaCTL3-depleted fat body (Fig 4G). RT-qPCR analysis of randomly selected *β-1*,*3-GRP 3*, *PGRP C*, *lebocin*, *attacin*, *pre-HP21*, *SPI 3/4*, *TIP-like*, and *ECSIT* further validated RNA-seq results (S6A–S6H Fig).

The overall decreased expression of AMPs was consistent with decreased antibacterial activity of the plasma in HaCTL3-depleted samples. To test whether HaCTL3-controlled AMPs were involved in defending against *E*. *mundtii*, we expressed and purified recombinant attacin (rAtt), lebocin (rLeb), and gloverin (rGlo) (Fig 4H). All of these recombinant proteins inhibited the growth of *E*. *mundtii* (Fig 4I). Hence, we concluded that not only HaCTL3 but also HaCTL3-regulated AMPs contribute to *E*. *mundtii* elimination and suppression in the hemolymph of *H*. *armigera*.

### Hemolymph *E*. *mundtii* comes from the gut, and HaCTL3 contributes to limiting *E*. *mundtii*

#### *E*. *mundtii* can escape from the gut to the hemolymph

**Fig 5:**
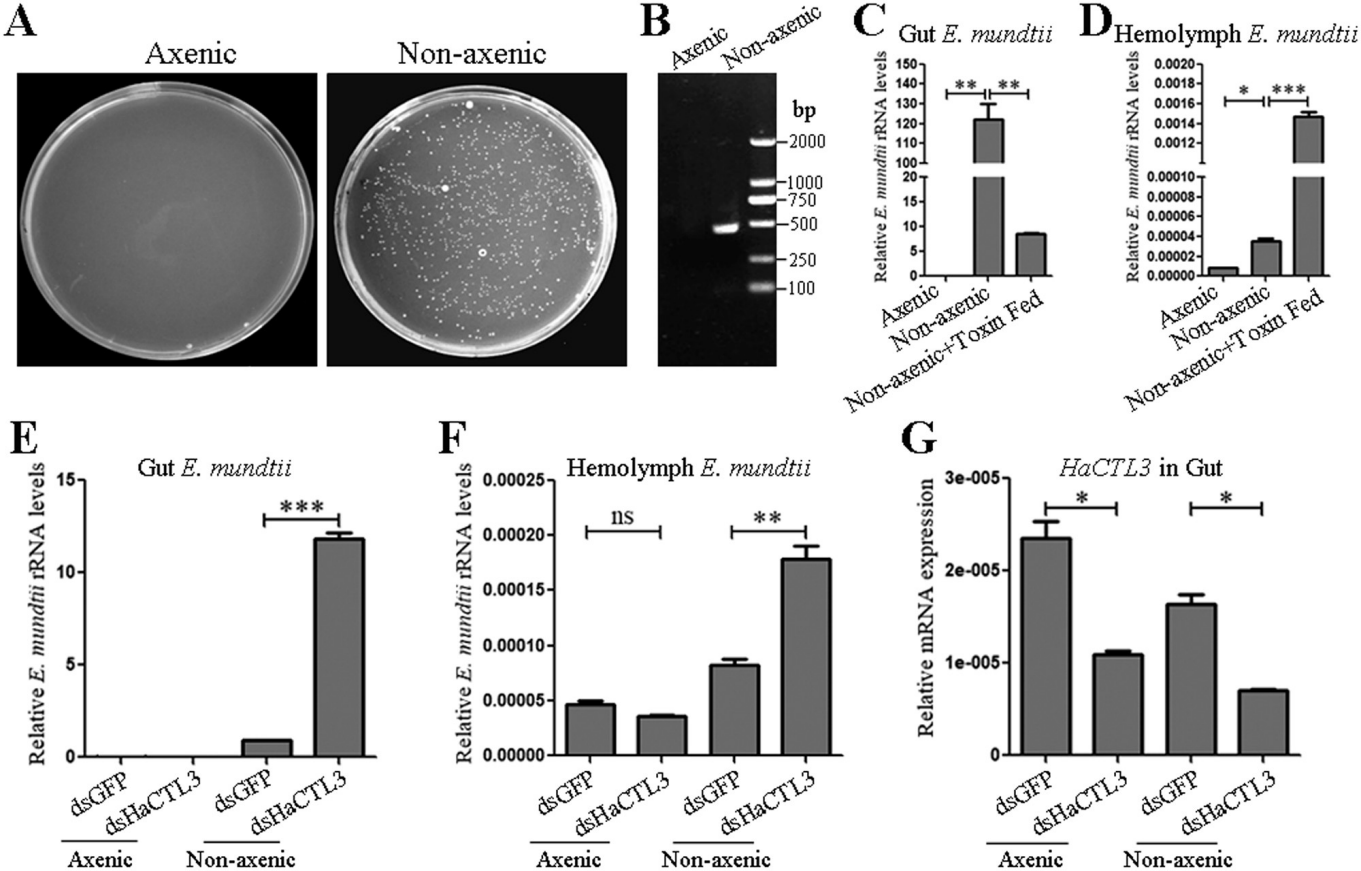
HaCTL3 depletion leads to increased load of *E*. *mundtii* in the gut and hemolymph. (A, B) Confirmation of elimination of gut bacteria by culturing gut homogenates on LB agar plates (A), and by conducting PCR analysis on gut homogenates using universal primers (16S-F and 16S-R) of bacterial 16S rRNA gene (B). (C, D) Quantification of gut *E*. *mundtii* (C) and hemolymph *E*. *mundtii* (D) in axenic and nonaxenic larvae, as well as *B*. *thuringiensis* toxin-fed larvae. (E, F) Quantification of gut *E*. *mundtii* (E) and hemolymph *E*. *mundtii* (F) in axenic and nonaxenic larvae treated with dsHaCTL3 or dsGFP. Quantification was by qPCR analysis using primers of *E*. *mundtii*-specific 16S rRNA gene. (G) RT-qPCR analyses confirming the depletion of *HaCTL3* transcripts in gut. The bar represents mean ± SEM from three biological replicates. *0.01 < *p* < 0.05, **0.001 < *p* < 0.01, ***p < 0.001 (Student’s *t*-test).

*Enterococcus* species are ubiquitous members of the normal gut microbiota of lepidopteran larvae [42]. To investigate whether *E*. *mundtii* in the hemolymph originated from the gut of *H*. *armigera*, we obtained axenic larvae via treatment with oral antibiotics. The efficiency of gut bacteria elimination was confirmed by plating gut homogenates onto LB agar plates (Fig 5A) and performing PCR analysis using universal primers F and R of bacterial 16S rRNA gene (Fig 5B). Quantitative PCR (qPCR) analysis was performed using *E*. *mundtii*-specific 16S rRNA gene PCR primers (S6 Table). The results indicated that the load of gut *E*. *mundtii* could hardly be detected in axenic larvae, whereas the load of gut *E*. *mundtii* in nonaxenic larvae was much higher (Fig 5C). Consistent with this, the load of hemolymph *E*. *mundtii* in axenic larvae was also much lower than that in nonaxenic larvae, suggesting that *E*. *mundtii* can escape from the gut to the hemolymph (Fig 5D). For a positive control, we treated larvae with the *B*. *thuringiensis* toxin, which binds receptors on the gut epithelium and leads to pore formation [43]. Quantitative PCR showed that the load of gut *E*. *mundtii* largely decreased, while the load of hemolymph *E*. *mundtii* exhibited the opposite trend after treatment of toxin (Fig 5C and 5D).

#### Depletion of HaCTL3 results in an increase of *E*. *mundtii* load in the gut and hemolymph

To confirm that HaCTL3 is involved in maintaining a low abundance of *E*. *mundtii*, we knocked down HaCTL3 in either axenic larvae or nonaxenic larvae and quantified *E*. *mundtii* by qPCR. The load of gut *E*. *mundtii* in axenic larvae was hardly detectable whether or not HaCTL3 was depleted. The load of gut *E*. *mundtii* in nonaxenic larvae was largely increased after HaCTL3 depletion when compared with the control group (Fig 5E). Consistent with the trend in the gut, HaCTL3 depletion significantly increased the load of hemolymph *E*. *mundtii* in nonaxenic larvae. No significant differences arose in hemolymph *E*. *mundtii* load between dsHaCTL3- and dsGFP-injected axenic larvae (Fig 5F). Successful knockdown of HaCTL3 in the guts of axenic and nonaxenic larvae was confirmed (Fig 5G). These results indicate that HaCTL3 maintains the low abundance of *E*. *mundtii* in the gut and hemolymph, and *E*. *mundtii* in hemolymph originates from the gut.

### Injection of *E*. *mundtii* mimics the HaCTL3-depleted phenotypes

#### Depletion of HaCTL3 reduces larval body size in nonaxenic but not in axenic larvae

To associate HaCTL3-depleted phenotypes with gut-derived microbes, we compared larval growth between HaCTL3-depleted nonaxenic group and HaCTL3-depleted axenic group. As shown in S7A and S7B Fig, the length was significantly smaller and the weight was significantly reduced in dsHaCTL3-injected nonaxenic larvae compared with that of dsHaCTL3-injected axenic larvae. Further, nonaxenic larvae exhibited significantly smaller length and significantly reduced weight at 72 h post-dsHaCTL3 injection compared with that of post-dsGFP injection, consistent with Fig 1B and 1C. However, axenic larvae showed no significant differences in larval length and weight between the treatment of dsHaCTL3 and dsGFP. These results clearly indicated that it was gut-derived microbes that resulted in HaCTL3-depleted body size.

#### Injection of *E*. *mundtii* into the larval hemocoel reduces body size and delays pupation time

**Fig 6:**
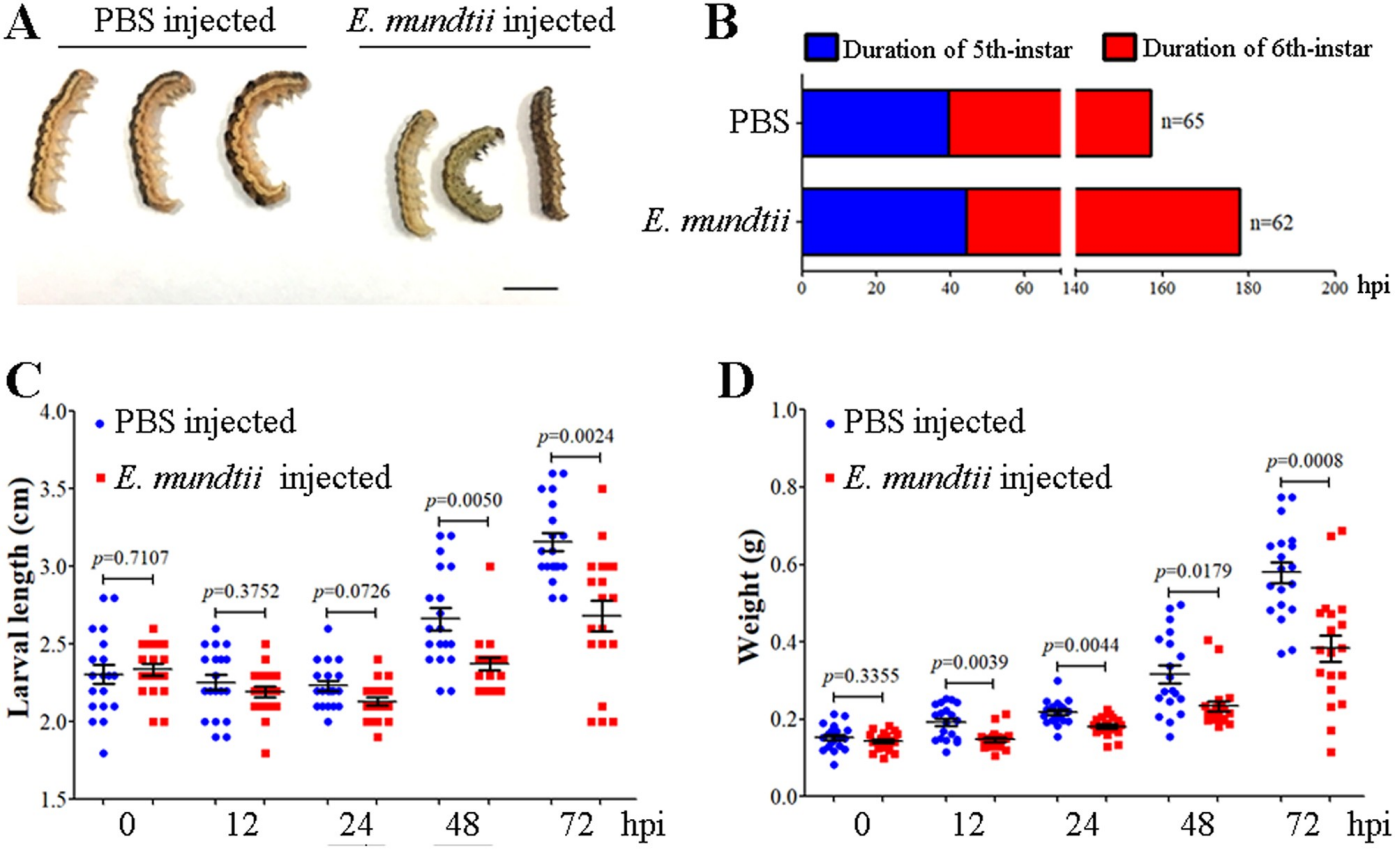
Reduced body size and delayed pupation in *E*. *mundtii*-injected larvae. (A) The size of representative *E*. *mundtii*- or PBS-injected larvae. The larvae were photographed at 42 h post-*E*. *mundtii* injection (hpi). Bar = 0.5 cm. (B) Prolonged duration of fifth- and sixth-instar in *E*. *mundtii*-injected larvae. The duration of fifth- and sixth-instar was measured based on 62 *E*. *mundtii*-injected and 65 PBS-injected larvae, respectively. (C, D) Larval body length (C) and weight (D) were reduced in *E*. *mundtii*-injected larvae. Larval body length and weight were measured at 0, 12, 24, 48, and 72 hpi. The *p* value was calculated by the Student’s *t* test for paired samples, and a *p* value of < 0.05 was considered statistically significant.

Because HaCTL3 depletion results in a significant increase of *E*. *mundtii* abundance in larval hemolymph, we reasoned that hemolymph *E*. *mundtii* originated from the gut may be the cause of the HaCTL3-depleted phenotypes. To test this, we injected *E*. *mundtii* into the larval hemocoel, and a visibly reduced body size was observed when compared with the control group (Fig 6A). The duration of fifth-instar is approximately 5 h longer on average in *E*. *mundtii*-injected larvae than that in the control group. *E*. *mundtii*-injected sixth-instar larvae also showed delayed pupation for an average of approximately 16 h relative to the control group (Fig 6B). Larvae exhibited significantly smaller length at 48 and 72 h post-*E*. *mundtii* injection (Fig 6C). Larvae also exhibited significantly reduced weight at 12, 24, 48, and 72 h post-*E*. *mundtii* injection (Fig 6D).

#### Injection of *E*. *mundtii* into the larval hemocoel suppresses 20E and JH signaling

To test whether ecdysone or JH is responsible for the reduced body size and delayed pupation, we compared the levels of these two hormones between *E*. *mundtii*-injected and PBS-injected larvae. Ecdysone titres and genes involved in 20E signaling were tested at the wandering stage, whereas JH titres and the genes involved in JH signaling were determined at the feeding stage of sixth-instar larvae.

**Fig 7:**
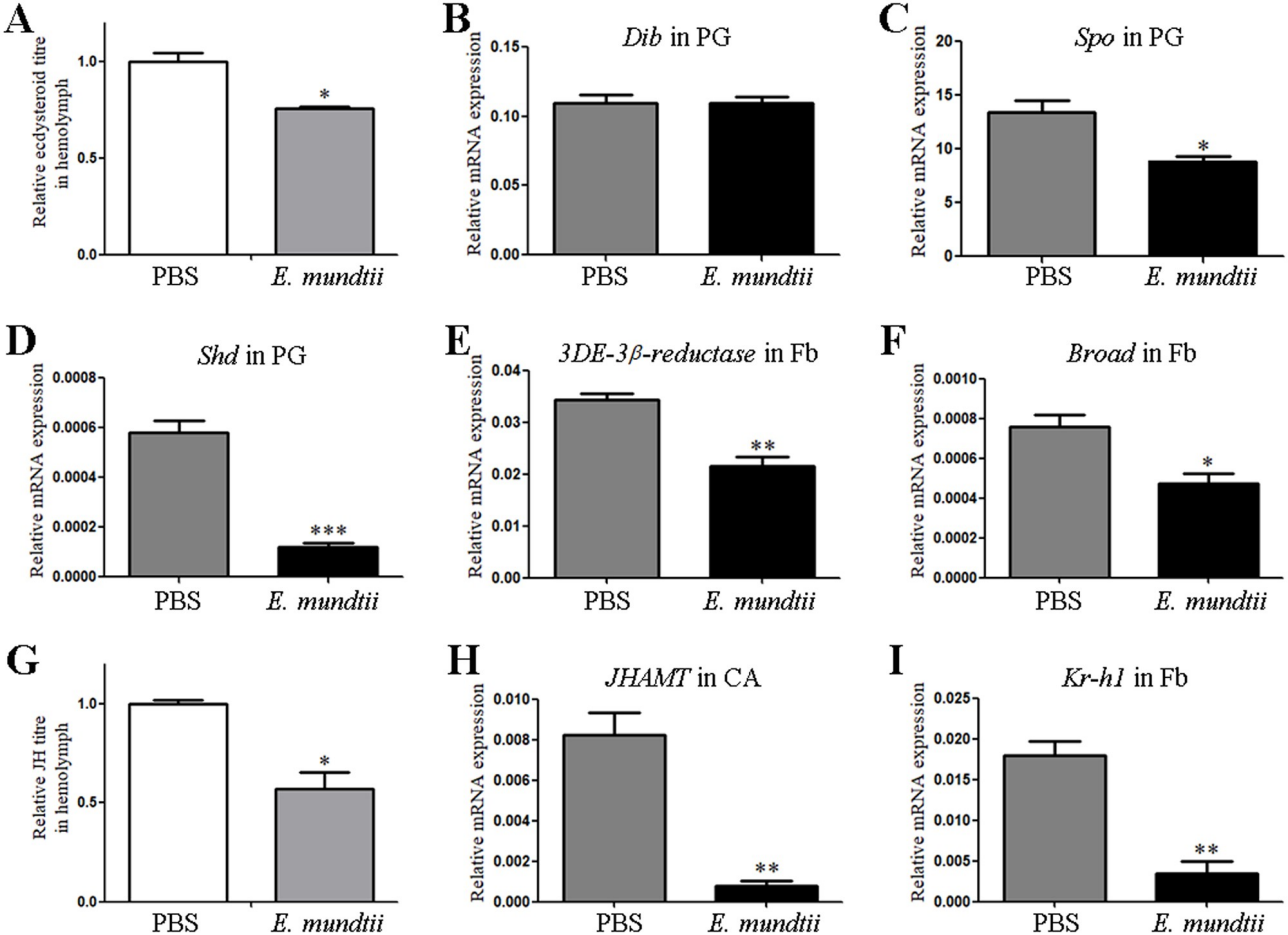
Suppression in 20E and JH signaling in *E*. *mundtii*-injected larvae. (A, G) Measurement of relative ecdysone titres (A) and JH titres (G) in the hemolymph of *E*. *mundtii*- or PBS-injected larvae. (B–F, H, I) Expression profiles of *Dib* (B), *Spo* (C), and *Shd* (D) in PG, *3DE-3β-reductase* (E), *Broad* (F), and *Kr-h1* (I) in Fb, and *JHAMT* (H) in CA of *E*. *mundtii*-injected and PBS-injected larvae. PG, prothoracic gland; Fb, fat body; CA, corpora allata. The bar represents mean ± SEM from three biological replicates. *0.01 < *p* < 0.05, **0.001 < *p* < 0.01, ***p < 0.001 (Student’s *t*-test).

Hemolymph ecdysone titres were significantly lower in *E*. *mundtii*-injected larvae than those of the control group (Fig 7A). As shown in Fig 7B–7D, *Spo* and *Shd* exhibited decreased expression in the PG of *E*. *mundtii*-injected larvae, whereas no significant differences of *Dib* expression were detected. The expression of *3DE-3β-reductase* was downregulated in the fat body of *E*. *mundtii*-injected larvae (Fig 7E). The expression of *Broad* was also decreased in the fat body of *E*. *mundtii*-injected larvae (Fig 7F). These results suggest that 20E signaling in *E*. *mundtii*-injected larvae was suppressed, which was consistent with the results in HaCTL3-depleted samples.

Likewise, hemolymph JH titres were significantly lower in *E*. *mundtii*-injected larvae than those in the control group (Fig 7G). The expression of *JHAMT* in the CA was downregulated in *E*. *mundtii*-injected larvae relative to the control groups (Fig 7H). *Kr-h1* also exhibited lower expression in the fat body of *E*. *mundtii*-injected larvae (Fig 7I). These results suggested that JH signaling in *E*. *mundtii*-injected larvae was suppressed, which also was consistent with the results for the HaCTL3-depleted samples.

Hence, we concluded that the reduced body size and delayed pupation in HaCTL3-depleted larvae is at least partly due to the increased abundance of *E*. *mundtii* in the hemolymph.

## Discussion

**Fig 8:**
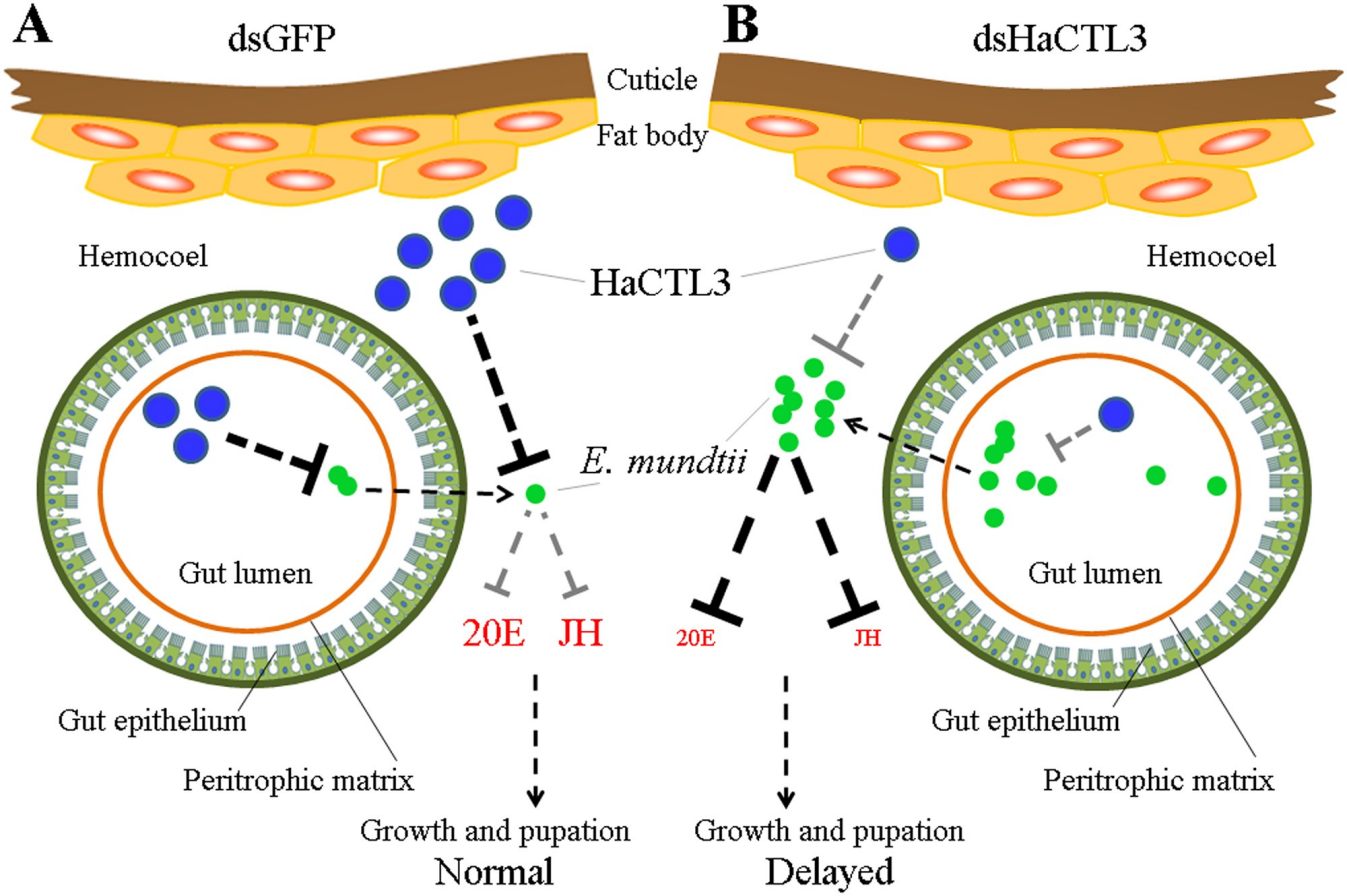
Model of the influence of HaCTL3 on *H*. *armigera* larval growth and pupation. (A) HaCTL3 promotes clearance of *E*. *mundtii*, resulting in low abundance of *E*. *mundtii* in the gut and hemolymph. Low abundance of *E*. *mundtii* in hemolymph cannot suppress 20E and JH signaling, resulting in normal growth and pupation. (B) Depletion of HaCTL3 weakens the ability to clear *E*. *mundtii*, resulting in high abundance of *E*. *mundtii* in the gut and hemolymph. High abundance of *E*. *mundtii* in hemolymph suppresses 20E and JH signaling, thus leading to reduced body size and delayed pupation.

An intriguing finding in the current study is that CTL as a PRR was able to modulate insect development. Most studies to date focus on endocrine regulation of immunity [4–11]. However, research on immune regulation of the endocrine system and development is quite limited. The use of larger lepidopterans for research has the advantage of significant developmental changes. Here, we used *H*. *armigera* as a model and found that HaCTL3 regulates the insect endocrine system and development through modulation of *E*. *mundtii* in the hemolymph. As summarized in Fig 8, HaCTL3 and its regulated AMPs in control larvae play important roles in the direct phagocytosis and eradication of *E*. *mundtii*, thus maintaining low levels of *E*. *mundtii* in the hemolymph. The low abundance of *E*. *mundtii* cannot suppress hormonal signaling; thus, larvae grow normally. However, depletion of HaCTL3 increases the load of *E*. *mundtii* in the hemolymph, which may escape from the gut. The dominance of *E*. *mundtii* in the hemolymph suppresses hormonal signaling, thus resulting in reduced body size and delayed pupation. This study may therefore represent a novel mechanism of immune system regulation of the endocrine system and will be beneficial for our understanding of immune-endocrine interaction.

### Presence of hemolymph microbiota in *H*. *armigera*

Conventional wisdom holds that the hemolymph of healthy insects is sterile. However, increasing evidence indicates that the hemolymph of some apparently healthy insects harbors various bacteria such as Enterobacteriaceae [17]. Various bacteria also are present in the hemolymph of some apparently healthy aquatic invertebrates [44]. In this study, we found that various bacteria colonized the hemolymph of apparently healthy *H*. *armigera*, with much higher diversity and much more abundance at the feeding stage than that at the wandering stage. These results were consistent with the enhanced antibacterial activity of the plasma and increased expression levels of PRRs and AMPs during the wandering stage relative to the feeding stage, as has been demonstrated in our previous results [9]. The persistence of microbiota in the hemolymph of *H*. *armigera* may arise from bacterial evasion or tolerance of insect immunity. The microbiota may function as a constant challenge to the immune system and generate basal immunity, which would not only restrict symbiotic bacteria but also resist external pathogens, as has been speculated for aquatic invertebrates [44]. Two dominant families of bacteria in the hemolymph during the wandering stage, namely Acetobacteraceae and Lactobacillaceae, may interact with hormonal signaling and be involved in modulating *H*. *armigera* development, based on the studies of gut symbiotic bacteria in *Drosophila* [13, 14].

### HaCTL3 shapes microbiota and maintains low levels of hemolymph *E*. *mundtii*

Many studies have suggested that the host immune system plays crucial roles in shaping the gut microbiota [45]. Mosquito PGRP-LC limits gut microbiota by activating the immune-deficiency pathway [27]. Mosquito CTLs coat the bacterial surface and counteract AMP activity, thus facilitating bacterial colonization [28]. However, molecular players involved in shaping hemolymph microbiota are mostly identified in aquatic invertebrates but rarely in insects. Lysozyme silencing in the shrimp largely increases the number of hemolymph bacteria [46]. Depletion of a shrimp CTL leads to high host mortality as a result of increased bacterial counts in the hemolymph [29]. The wide spectrum of bacteria recognized by HaCTL3 has determined its critical role. The suppression of bacterial clearance capacity in the hemocoel of HaCTL3-depleted larvae may be attributable to compromised hemocytic phagocytosis and plasma antibacterial activity. This further supported that HaCTL3 is a central regulator in restricting microbiota proliferation and achieving homeostasis in the hemolymph. Depletion of HaCTL3 resulted in a decrease of almost all PRR and AMP transcripts, which may explain why depletion of HaCTL3 led to a greater abundance of hemolymph microbiota. Although it remains unclear how HaCTL3 controlled expression of the PRRs and AMPs, some suggest that CTLs could regulate the expression of AMPs via the JAK/STAT pathway [47, 48].

Depletion of HaCTL3 increased the load of *E*. *mundtii* in both the gut and hemolymph, suggesting the involvement of HaCTL3 in maintaining a low level of *E*. *mundtii*. As HaCTL3 is mainly expressed in the fat body and is subsequently secreted into hemolymph [32, 34], soluble HaCTL3 may directly target *E*. *mundtii* and employ hemocytes to migrate toward it for phagocytosis. Furthermore, AMPs (attacin, lebocin, and gloverin) controlled by HaCTL3 exhibited direct bacteriostatic activity toward *E*. *mundtii*, consistent with the involvement of several AMPs in the suppression of hemolymph microbiota [29, 49].

### High abundance of hemolymph *E*. *mundtii* leads to reduced body size and delayed pupation

Given that *E*. *mundtii* is usually the dominant symbiotic bacterium in the lepidopteran gut [50, 51], we reasoned that *E*. *mundtii* detected in the *H*. *armigera* hemolymph originated from the gut. This speculation was demonstrated to some extent by the significant increase in the load of hemolymph *E*. *mundtii* in nonaxenic but not in axenic larvae in response to HaCTL3 depletion. Moreover, significantly reduced body size was observed in HaCTL3-depleted nonaxenic larvae when compared with that of HaCTL3-depleted axenic larvae, clearly associating the reduced body size in HaCTL3-depleted larvae with gut microbes but not others. Given that depletion of HaCTL3 increased the load of hemolymph *E*. *mundtii*, and injection of *E*. *mundtii* into hemocoel reduced larval body size and delayed pupation, we believed that *E*. *mundtii* were such critical microbes that translocated from the gut to the hemolymph. Many factors such as bacterial or fungal chitinolytic enzymes can increase the permeability of peritrophic matrix [52, 53]. The escape of *E*. *mundtii* from the gut may be associated with dysbiosis of gut microbiota caused by HaCTL3 depletion and the subsequent destruction of structural integrity of the peritrophic matrix, as proposed previously [54]. Interestingly, genes encoding peritrophin and mucin, the important components for the structure and function of peritrophic matrix [55, 56], increased their transcripts in HaCTL3-depleted fat body (S3 Table). However, whether these genes are involved in translocation of *E*. *mundtii* is still under exploration.

Despite the vast majority of studies regarding gut commensal bacteria regulation of host immunity and metabolism [57–59], information about the roles of insect hemolymph microbiota remains obscure. In addition to serving as the constant stimulator of host basal immunity, some hemolymph symbionts may benefit the host by resisting certain kinds of pathogens. For example, the facultative endosymbiont *Regiella insecticola* in the hemolymph of the aphid enhance host resistance to the fungal pathogen [15]. Others have revealed that hemolymph microbes became opportunistic pathogens upon translocation from the gut. For example, *Enterococcus faecalis* in *Manduca sexta* completes its commensal-to-pathogen switch after migration of bacteria from the gut to the hemocoel [20]. Plant-mediated translocation of bacteria from the gut to the hemolymph reduces larval growth and elevates mortality of the fall armyworm [19]. *E*. *mundtii* has demonstrated previously to be a pathogenic agent for the flacherie disease of the silkworm larvae, with symptoms involving retarded growth, flaccidity, and cessation of feeding [60]. In the present study, reduced larval body size and delayed pupation were observed in *H*. *armigera* following direct injection of *E*. *mundtii* into the hemocoel, which may be partly due to suppressed ecdysone and JH hormonal signaling. Administration of 20E and/or JH rescues larval body size and pupation, suggesting endocrinological changes caused by *E*. *mundtii* are responsible for HaCTL3-depleted phenotypes. *E*. *mundtii* could produce trypsins and express serine- and cysteine-proteinase activities, with the ability to metabolize carbon sources, nitrogen sources, phosphorus sources and sulfur sources [61–63]. It was suggested that the effectors produced by *E*. *mundtii* might interact with hormonal signaling and contribute to these observed phenotypes. Gut bacterial effectors such as acetic acid produced by *A*. *pomorum* promote insulin signaling and modulate insect host development [13]. Functional annotation of the *E*. *mundtii* genome indicates remarkable features related to lactic acid fermentation and *E*. *mundtii* is capable of producing lactic acid efficiently [64]. However, the specific and mechanistic effectors that result in the observed phenotypes still need to be characterized.

### Homeostasis between hemolymph *E*. *mundtii* and *H*. *armigera* mediated by HaCTL3

Despite great repertoires of immune molecules in invertebrates, due to the energy-consuming nature of the immune response, host selectively expresses a small number of immune molecules to fight infection. HaCTL3 as a PRR with a broad recognition spectrum is one such critical molecular player, not only in defending against parasitic nematodes [34], but also participating in the homeostasis between host and hemolymph microbiota. Because depletion of HaCTL3 significantly increased the *E*. *mundtii* load in the gut, we cannot exclude the possibility that dysbiosis of gut microbiota may influence the development of *H*. *armigera*. However, given that injection of *E*. *mundtii* into the hemocoel mimics HaCTL3-depleted phenotypes, we conclude that the reduced body size and the delayed pupation caused by HaCTL3 depletion is at least partly due to the increased abundance of *E*. *mundtii* in the hemolymph. Therefore, our study provides novel insights into the mechanism employed by immunity to influence development.

## Materials and methods

### Insect rearing, antibiotic treatment, and toxin feeding

*H*. *armigera* larvae were maintained at 28°C ± 1°C, 70% relative humidity, and 14 h light/10 h dark photoperiod. For the nonaxenic group, larvae were fed a sterilized artificial diet mainly comprised of soybean powder and wheat germ. For axenic treatment, larvae were reared with the same artificial diet supplemented with penicillin (10 unit/mL), gentamicin (15 μg/mL), and streptomycin (10 μg/mL) from the time of the third-instar. For toxin treatment, fifth-instar larvae were fed the commercial *B*. *thuringiensis* toxin Cry1Ac incorporated into a normal artificial diet, with 10 μg toxin per g diet. Gut and hemolymph were collected from nonaxenic, axenic, and toxin-fed larvae at the sixth-instar at 24 h PE for bacterial genomic DNA extraction.

To confirm the complete elimination of bacteria, sixth-instar larvae at 24 h PE were surface-sterilized in 75% ethanol and rinsed three times with sterile PBS (pH 7.4). Guts were dissected from axenic and nonaxenic larvae, rinsed with sterile PBS, and homogenized. Homogenates were plated onto LB agar plates and incubated at 37°C overnight. PCR analyses of gut homogenates were performed using universal primers (16S-F and 16S-R) of the bacterial 16S rRNA gene.

### RNAi

We performed RNAi according to the method described previously [34]. Briefly, two dsRNAs of *HaCTL3* (namely, dsHaCTL3 and dsHaCTL3-2) were synthesized based on non-overlapping sequences using a MEGAscript kit (Ambion, Austin, TX, USA). The *GFP* gene was used to produce control dsRNA (dsGFP). Primers used for dsRNA generation are listed in S6 Table. Each fourth-instar larva (either axenic or nonaxenic) at 6–12 h PE was injected with 5 μL of the corresponding dsRNA (1.0 μg/μL). Same amount of dsGFP was used as a control. Hemolymph, fat bodies, guts, PG, and CA were collected at either 96 h (feeding) or 144 h (wandering) post-dsRNA injection for further assays.

### Hemolymph microbiota collection, 16S rRNA sequencing, and bioinformatics analysis

We prepared four larval groups, namely sixth-instar larvae at 24 h PE (feeding) and 96 h PE (wandering), as well as larvae at 144 h post-dsHaCTL3 or -dsGFP treatment. Each group contained three biological replicates, and each replicate contained pooled hemolymph samples from six larvae. Prior to dissection, larvae were surface-sterilized. Hemolymph was collected from each group by cutting the abdominal feet of larvae without touching guts and diluted threefold in sterile anticoagulant buffer. After centrifugation at 1,000 g for 10 min, the precipitation (containing hemocytes and microbiota) was harvested and used for extraction of total metagenomic DNA. Subsequently, the V3 and V4 hypervariable regions of the 16S rRNA gene pool were amplified with the primers 338F and 806R (S6 Table). PCR amplicon libraries were then generated and subjected to high-throughput sequencing on an Illumina MiSeq PE300 platform (Illumina, San Diego, CA, USA) at Majorbio Bio-Pharm Technology Co., Ltd. (Shanghai, China).

Raw sequences of each sample were processed to obtain clean data according to the method described previously [65]. OTU generation and clustering were performed with a cut-off of 97% similarity using USEARCH [66]. Phylogenetic affiliation of each 16S rRNA gene sequence was analyzed against the SILVA 16S rRNA database (http://www.arb-silva.de) with a confidence threshold of 70%. Differential analyses of the relative abundance of hemolymph microbiota between groups were performed at the family level.

### Isolation, identification, and inoculation of *E*. *mundtii*

To isolate bacteria from the hemolymph, each larva was surface-sterilized, and hemolymph was collected from sixth-instar larvae at 144 h post-dsHaCTL3 injection. Hemolymph was diluted (1:3) in sterile anticoagulant buffer and spread onto LB agar plates. After incubation at 37°C overnight, several morphologically distinct colonies were visible on the LB agar plates. Genomic DNA was extracted from different bacterial isolates using bacterial DNA kit (Omega Bio-Tek, Norcross, GA, USA), and a DNA fragment was amplified using universal 16S rRNA gene primers 27F and 1492R (S6 Table). After sequencing and blasting against GenBank database, we finally isolated and characterized the gram-positive bacteria *E*. *mundtii*.

*E*. *mundtii* in mid-logarithmic phase was collected and suspended in sterile PBS at 2 × 10^8^ cells/mL. Each fifth-instar larva at 6–12 h PE was injected with 5 μL of bacterial suspension. Hemolymph, fat bodies, PG, and CA were collected at either 72 h (feeding) or 120 h (wandering) post-*E*. *mundtii* injection for further assays.

### Measurement of larval length, larval weight, and developmental timing

The assay of larval length and weight was based on 24 dsHaCTL3- or dsGFP-injected larvae, as well as 21 HaCTL3-2- or dsGFP-injected larvae. We individually measured larval length and weighed larvae every 24 h until at 144 h post injection (hpi). We also measured the length and weight of 35 dsHaCTL3- or dsGFP-injected nonaxenic larvae, as well as 35 dsHaCTL3- or dsGFP-injected axenic larvae at 0 and 72 hpi. To evaluate the effect of *E*. *mundtii* on larval length and weight, 19 *E*. *mundtii*- or PBS-injected larvae were analyzed at 0, 12, 24, 48, and 72 hpi.

To measure the mean duration of the fourth-, fifth-, and sixth-instars, we recorded each individual instar every 1 h from the beginning of injection until pupation. A total of 47 dsHaCTL3- or dsGFP-injected larvae, 26 dsHaCTL3-2- or dsGFP-injected larvae, 62 *E*. *mundtii*-injected larvae, and 65 PBS-injected larvae were analyzed.

### Measurement of ecdysone and JH titres, and hormonal treatment of larvae

Hemolymph collected at 96 h post-dsRNA injection or at 72 h post-*E*. *mundtii* injection was used to determine JH titres, while hemolymph collected at 144 h post-dsRNA injection or 120 h post-*E*. *mundtii* injection was used to measure ecdysone titres, as previously described [67–69]. For ecdysone measurement, hemolymph was collected from pools of five larvae in each treatment group, and 50 μL of hemolymph was diluted fivefold with chilled methanol. Samples were vortexed and centrifuged for 10 min at 12,000 g, and the upper layer was transferred to a clean tube and dried by vacuum centrifugation. After resuspension in enzyme immunoassay (EIA) buffer, the sample was subjected to EIA kit (Cayman Chemical, Ann Arbor, MI, USA) according to the manufacturer’s instructions. For JH measurement, hemolymph was collected from pools of five larvae per treatment. After vortex and centrifugation, the organic phase was transferred to a new tube, dried, and subsequently resuspended in acetonitrile. Quantification of JH titres was performed by liquid chromatography-tandem mass spectrometry.

We injected each fourth-instar larva at 6–12 h PE with 5 μL of dsHaCTL3 (1.0 μg/μL). Then these larvae were divided into four groups, and each group was injected with JH III at 500 ng/5μL per larva, or 20E at 500 ng/5μL per larva, or both. JH III was injected at 12 h PE, whereas 20E was injected at 72 h PE of sixth-instar. The equivalent amount of DMSO as solvent control was injected at the same stage. A total of 33 JH III- or solvent-injected larvae were analyzed at 0 and 48 hpi to evaluate the effect of JH on HaCTL3-depleted larval length and weight. A total of 23 JH III-, 20E-, JH III- plus 20E-, or solvent-injected larvae were used to measure the mean duration of the sixth-instar, as described above.

### Binding of rHaCTL3 to bacteria

We tested the ability of rHaCTL3 to bind bacteria as described previously [70]. Gram-positive bacteria (*E*. *mundtii*, *S*. *aureus*, *M*. *pumilum*, and *C*. *funkei*) and gram-negative bacteria (*E*. *coli*, *V*. *anguillarum*, and *O*. *cytisi*) were used for testing. Briefly, bacteria were resuspended in TBS (500 μL) to an OD_600_ of 1.0, and rHaCTL3 (1.0 mg/mL, 200 μL) was subsequently added. After incubation for 30 min in the presence or absence of 10 mM CaCl_2_, bacteria were collected, washed with TBS four times, and eluted with 500 mM NaCl and 7% SDS, sequentially. Samples were then subjected to western blot analyses.

It was hypothesized that components of the bacterial cell wall were responsible for binding. Hence, eight saccharides (D-trehalose dihydrate, LPS, D-galactose, sucrose, D-maltose monohydrate, PGN, D-mannose, and D-glucose) were applied to test their ability to inhibit the binding of rHaCTL3 to *S*. *aureus*. Each carbohydrate (1.0 mg/mL, 50 μL) was preincubated with rHaCTL3 (1.0 mg/mL, 50 μL), and 500 μL of TBS containing *S*. *aureus* (2 × 10^8^ cells/mL) was added. After incubation, bacterial cells were pelleted, washed, eluted, and analyzed by western blot.

### Western blot

Protein samples collected from RNAi treatments were quantified and applied for western blot analyses. Equal aliquots of protein samples were resolved via 12.5% SDS-PAGE and transferred to nitrocellulose membranes. Rabbit polyclonal antibody against HaCTL3 was produced previously [34] and used to detect the proteins. Rabbit polyclonal antibody against α-Tubulin (Earthox, San Francisco, CA, USA) was used as a loading control. Equal loading of plasma protein was further confirmed by Coomassie blue staining of the gels. Samples eluted from bacteria were also applied for western blot analyses, and anti-His antibody (TransGen Bio-tech, Beijing, China) was used to detect rHaCTL3.

### *In vivo* bacterial clearance assay

To determine whether HaCTL3 was involved in antibacterial activity, we performed *in vivo* bacterial clearance assay as described previously [71]. Briefly, larvae were injected with *E*. *coli* or *S*. *aureus* (2 × 10^7^ CFUs/larva) at 96 hpi of *HaCTL3* or *GFP* dsRNA (control). Hemolymph was collected and immediately diluted (1:3) in sterile anticoagulant buffer at 1 hpi of bacteria. Hemolymph-anticoagulant mixture (100 μL) was plated onto LB agar plates. After culturing at 37°C overnight, CFUs were counted in each plate.

### *In vitro* assay of antibacterial activity in plasma

To determine whether HaCTL3 influences the antibacterial activity of the plasma, hemolymph was collected from larvae at 96 hpi of *HaCTL3* or *GFP* dsRNA, and diluted (1:3) in sterile anticoagulant buffer. Cell-free plasma was then procured by centrifugation at 1,000 g for 10 min. Antibacterial activity was determined as previously described [9]. Briefly, 90 μL of plasma was mixed with a 10 μL suspension of *E*. *coli* or *S*. *aureus*. After incubation for 1 h at room temperature, the plasma-bacteria mixture was plated onto LB agar plates and cultured at 37°C overnight. CFUs in each plate were then counted.

### Phagocytosis assay

Fluorescein isothiocyanate (FITC, Sigma) labeling of *E*. *mundtii*, *S*. *aureus*, or *E*. *coli* was conducted at 37°C for 1 h as described previously [72]. FITC-labeled bacteria were washed five times with PBS and resuspended in PBS at a concentration of 2 × 10^8^ cells/mL.

Larvae were injected with FITC-labeled bacteria at 96 h post-dsHaCTL3 or -dsGFP injection. After 1 h, hemocytes were collected and spread onto slides. After fixing with 4% paraformaldehyde for 10 min, the hemocytes were observed under a fluorescence microscope. The phagocytic rate was defined as follows: [(number of hemocytes ingesting bacteria/the number of all hemocytes) × 100%]. Three replicates were performed, and six larvae were sacrificed for each assay.

To investigate whether rHaCTL3 promotes hemocytic phagocytosis, the FITC-labeled bacteria suspension as above was incubated with rHaCTL3. After adequately washing with PBS to remove redundant protein, bacteria with rHaCTL3 adhered were injected into the hemocoel of sixth-instar larvae at 24 h PE. Phagocytosis assays were performed as the same method described above.

### Transcriptome sequencing and bioinformatics pipeline

Total RNA from the fat bodies of larvae at 96 h post-dsHaCTL3 or -dsGFP injection was extracted using TRIzol (Invitrogen, Carlsbad, CA, USA). Six cDNA libraries (dsHaCTL3-1, -2, -3, dsGFP-1, -2, and -3) for Illumina sequencing were prepared. Briefly, mRNAs were enriched via magnetic beads and subsequently fragmented into short sequences. First-strand cDNAs were synthesized with SuperScript II reverse transcriptase, followed by synthesis of second-strand cDNAs using DNA polymerase I. After end repair, adaptor ligation, and further purification, cDNA libraries were obtained and sequenced on an Illumina Hiseq 2500 instrument at the Beijing Genomics Institute (Shenzhen, China).

After filtration of raw reads by removing adapters, low-quality reads, and unknown biases, clean reads were obtained. Trinity was used to assemble clean reads into contigs, which were further assembled into unigenes. All unigenes were run in BLASTx searches against the nonredundant database, with an e-value threshold of 10^−5^. Based on the BLASTx results, GO annotation for each unigene was conducted using Blast2GO. Clean reads were mapped into the unigenes by using Bowtie2, and expression levels of unigenes were quantified by using RNA-seq by expectation-maximization software [73]. DEGs between libraries were screened using DESeq2 [74] and PossionDis [75] with a cutoff of fold-change ≥ 2 and adjusted Pvalue (Padj) ≤ 0.05. DEGs were then annotated by GO with Bonferroni’s correction of *p* value ≤ 0.05.

### RT-qPCR

Total RNA of fat bodies, PG, CA, and guts either from dsRNA injection or from *E*. *mundtii* injection were extracted using TRIzol (Invitrogen). After treatment with DNase I, first-strand cDNA was synthesized with 2 μg of total RNA using EasyScript cDNA synthesis SuperMix (TransGen Bio-tech). RT-qPCR was performed with TransStart Top Green qPCR SuperMix (TransGen Bio-tech). Each sample was assayed in triplicate, and the relative expression for each gene was calculated using the 2^−Δ*CT*^ method (Δ*C*_*T*_ = *C*_*T*, tested gene_ −*C*_*T*, Haβ-actin_). Primers designed for RT-qPCR analysis are listed in S6 Table.

### Expression, purification, and antibacterial activity assay of AMPs

We expressed and purified rAtt, rLeb and rGlo as described previously [32]. Briefly, DNA fragments encoding Att, Leb, or Glo were subcloned into the pET32a vector and subsequently transformed into *E*. *coli* BL21 (DE3) competent cells. Primers designed for amplification of DNA fragments are listed in S6 Table. Expression of rAtt, rLeb, and rGlo was induced by isopropyl β-D-1-thiogalactopyranoside at a final concentration of 0.5 mM. After purification using High-Affinity Ni-NTA Resin (GenScript, Piscataway, NJ, USA), rAtt, rLeb, and rGlo were used for antibacterial activity assays. Empty pET32a vector as a control was transformed, and recombinant thioredoxin (rTrx, His-tagged) was induced and purified, according to the same method.

We conducted antibacterial activity assays against *E*. *mundtii* as described previously [71]. Bacterial suspension (2 × 10^5^ CFUs, 140 μL) cultured in LB medium was added to a 96-well polypropylene microtiter plate followed by addition of purified rAtt, rLeb, or rGlo (200 ng/μL, 40 μL) to each well. After incubation for 12 h at 28°C, bacterial growth was assayed by measuring the absorbance at 600 nm. Addition of rTrx was used as a control. Assays were performed in triplicate.

### Quantification of gut and hemolymph *E*. *mundtii* by qPCR

Genomic DNA from guts or hemolymph of axenic and nonaxenic larvae, as well as toxin-fed larvae was extracted using a genomic DNA isolation kit (Omega). Genomic DNA from the guts or hemolymph of axenic and nonaxenic larvae treated with *HaCTL3* or *GFP* dsRNA was also extracted. Quantification of *E*. *mundtii* by qPCR was conducted on genomic DNA using *E*. *mundtii*-specific 16S rRNA primers (S6 Table). Quantitative PCR was performed with a TransStart Top Green qPCR SuperMix (TransGen Bio-tech). Quantitative measurements were performed in triplicate and normalized against the housekeeping gene *β-actin* using the 2^−Δ*CT*^ calculation method.

## Supporting information

S1 Fig Reduced body size and delayed pupation by injection of another *HaCTL3* dsRNA (dsHaCTL3-2) into larval hemocoel. (A) RT-qPCR analysis showing *HaCTL3* depletion efficiency in fat body (Fb). The bar represents mean ± SEM from three biological replicates. *0.01 < *p* < 0.05 (Student’s *t*-test). (B) Western blot confirming the decreased expression of HaCTL3 proteins in Fb. (C, D) Larval body length (C) and weight (D) were reduced in HaCTL3-depleted larvae. Larval body length and weight were measured at 24, 48, 72, 96, 120, and 144 h post-dsRNA injection (hpi). The *p* value was calculated by the Student’s *t* test for paired samples, and a *p* value of < 0.05 was considered statistically significant. (E) Delayed pupation time in HaCTL3-depleted larvae. The duration of fourth-, fifth-, and sixth-instar was measured based on 26 individuals of each kind of treatment.
(TIF)

S2 Fig Rescued phenotypes of HaCTL3-depleted larvae upon 20E and/or JH treatment. (A, B) JH treatment elevates body length (A) and weight (B) in HaCTL3-depleted larvae. Larval body length and weight were measured at 0 and 48 h post injection (hpi) of JH or DMSO (as solvent control). The *p* value was calculated by the Student′s *t* test for paired samples, and a *p* value of < 0.05 was considered statistically significant. (C) JH or 20E plus JH treatment accelerates pupation of HaCTL3-depleted larvae. The duration of sixth-instar was measured based on 23 20E-, JH-, 20E plus JH-, or solvent-injected larvae pretreated with dsHaCTL3.
(TIF)

S3 Fig Venn diagram of OTU abundance in larval hemolymph. Hemolymph was collected from sixth-instar larvae at 24 h PE (6th-24 h; Feeding) and 96 h PE (6th-96 h; Wandering), as well as from larvae of dsHaCTL3- or dsGFP-injected (as control) groups.
(TIF)

S4 Fig HaCTL3 promotes bacterial clearance in the hemolymph. (A) Depletion of HaCTL3 suppresses bacterial clearance in the hemocoel of *H*. *armigera* larvae. Larvae pretreated with dsHaCTL3 or dsGFP were injected with *E*. *coli* or *S*. *aureus*. Hemolymph was collected at 1 h post-bacterial injection, and the number of CFUs was determined. (B) Binding of rHaCTL3 to *S*. *aureus*. *S*. *aureus* were incubated with rHaCTL3 for 30 min. After washing with TBS four times, *S*. *aureus* were pelleted and subjected to elution with 7% SDS. Lanes 1–4, TBS wash solution; lane 5, 7% SDS elution. (C) Binding of rHaCTL3 to various bacteria. Various bacteria were incubated with rHaCTL3, washed with TBS, and eluted with 7% SDS. (D) Inhibition analyses of the binding ability of rHaCTL3 to *S*. *aureus* by carbohydrates. Each carbohydrate was incubated with rHaCTL3 followed by addition of *S*. *aureus*. PGN exhibited a competitively inhibitory effect on the binding of rHaCTL3 to *S*. *aureus*. Samples were applied for detection by anti-His antibody. (E) rHaCTL3 promotes hemocytic phagocytosis of different bacteria. FITC-labeled *E*. *coli* or *S*. *aureus* were preincubated with rHaCTL3 or rTrx and then injected into the hemocoel of sixth-instar larvae at 24 h PE. (F) Depletion of HaCTL3 suppresses hemocytic phagocytosis. Larvae at 96 h post-dsHaCTL3 or dsGFP treatment were injected with FITC-labeled *E*. *coli* or *S*. *aureus*. (G) Depletion of HaCTL3 suppresses antibacterial activities in the plasma. Cell-free plasma was obtained from larvae pretreated with dsHaCTL3 or dsGFP. After incubation with *E*. *coli* or *S*. *aureus* for 1 h, the plasma-bacterial suspension was plated, and the number of CFUs was recorded. Columns represent the mean of three individual counts ± SEM. *0.01 < *p* < 0.05, **0.001 < *p* < 0.01 (Student’s *t*-test).
(TIF)

S5 Fig RNA-seq quantification analysis showing the global expression profile in response to HaCTL3 depletion. (A) Scatter plot represents DEGs in the HaCTL3-depleted fat body. DEGs were accepted with a cut-off of |log_2_FoldChange (dsHaCTL3/dsGFP)| ≥ 1 and Padj ≤ 0.05. (B) GO classification of DEGs in HaCTL3-depleted fat body. DEGs were assigned into three main GO terms, namely, biological process, cellular component, and molecular function, which were further subdivided into 30 subcategories. Asterisk represents that DEGs involved in immune system process were downregulated. (C-E) Heatmap showing that DEGs encoding PRRs (C), melanization-related proteins (D), and other immune-related proteins (E) varied in the HaCTL3-depleted fat body compared with that in the control sample, as characterized by RNA-seq analysis.
(TIF)

S6 Fig RT-qPCR confirming the expression of immune-related DEGs characterized by RNA-seq analysis. Randomly selected 2 *PRRs* (A, B), 2 *AMPs* (C, D), 2 *melanization-related* (E, F) and 2 other *immune-related* (G, H) DEGs were analyzed in response to HaCTL3 depletion. Error bars represent ±SEM. *0.01 < *p* < 0.05, **0.001 < *p* < 0.01, ***p < 0.001 (Student’s *t*-test).
(TIF)

S7 Fig Reduced body size in HaCTL3-depleted nonaxenic larvae compared with that in HaCTL3-depleted axenic larvae. (A, B) Larval body length (A) and weight (B) were significantly reduced in HaCTL3-depleted nonaxenic larvae when compared with that in HaCTL3-depleted axenic larvae, or compared with that in nonaxenic larvae treated with dsGFP. Larval body length and weight were measured in axenic and nonaxenic larvae treated with dsHaCTL3 or dsGFP at 0 and 72 h post-dsRNA injection (hpi). *0.01 < *p* < 0.05, **0.001 < *p* < 0.01, ***p < 0.001 (Student’s *t*-test).
(TIF)

S1 Table Hemolymph OTU taxonomy from larvae of feeding and wandering stages. (XLS)

S2 Table Hemolymph OTU taxonomy from dsHaCTL3-injected or dsGFP-injected larvae. (XLS)

S3 Table Differentially expressed genes (DEGs) in the HaCTL3-depleted fat body. (XLS)

S4 Table Gene ontology analysis of DEGs in the HaCTL3-depleted fat body. (XLS)

S5 Table Immune-related DEGs identified in the HaCTL3-depleted fat body. (XLS)

S6 Table Primers used in this study. (XLS)
