# Supplementary figures and images for: C-type lectin-mediated microbial homeostasis is critical for Helicoverpa armigera larval growth and development
Source: PLoS Pathog. 2020 Sep 30;16(9):e1008901. doi: 10.1371/journal.ppat.1008901 (PMC7549827; doi:10.1371/journal.ppat.1008901)

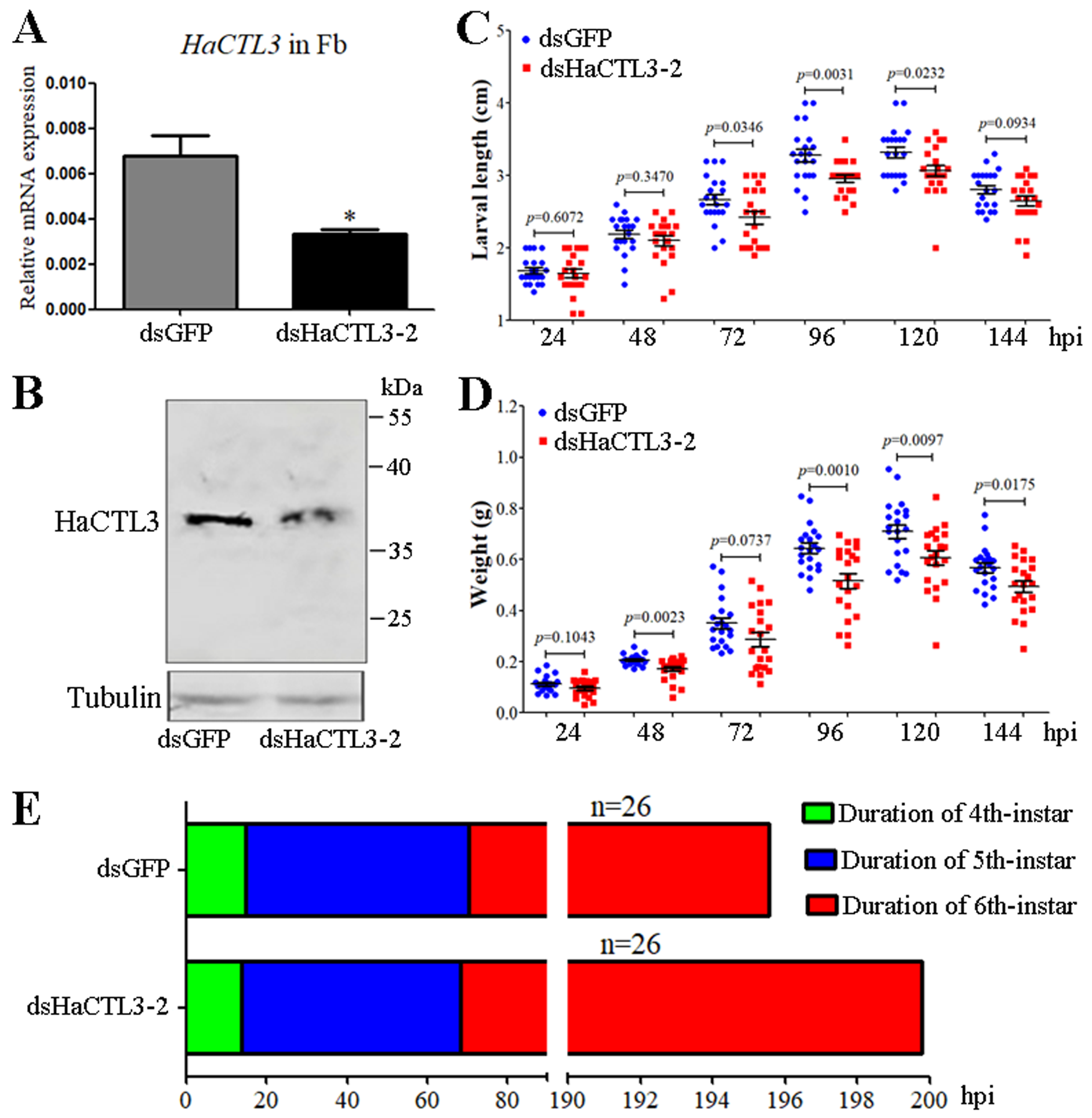

Supplement: S1 Fig — (A) RT-qPCR analysis showing HaCTL3 depletion efficiency in fat body (Fb). The bar represents mean ± SEM from three biological replicates. *0.01 < p < 0.05 (Student’s t-test). (B) Western blot confirming the decreased expression of HaCTL3 proteins in Fb. (C, D) Larval body length (C) and weight (D) were reduced in HaCTL3-depleted larvae. Larval body length and weight were measured at 24, 48, 72, 96, 120, and 144 h post-dsRNA injection (hpi). The p value was calculated by the Student’s t test for paired samples, and a p value of < 0.05 was considered statistically significant. (E) Delayed pupation time in HaCTL3-depleted larvae. The duration of fourth-, fifth-, and sixth-instar was measured based on 26 individuals of each kind of treatment. (TIF) [file ppat.1008901.s001.tif]

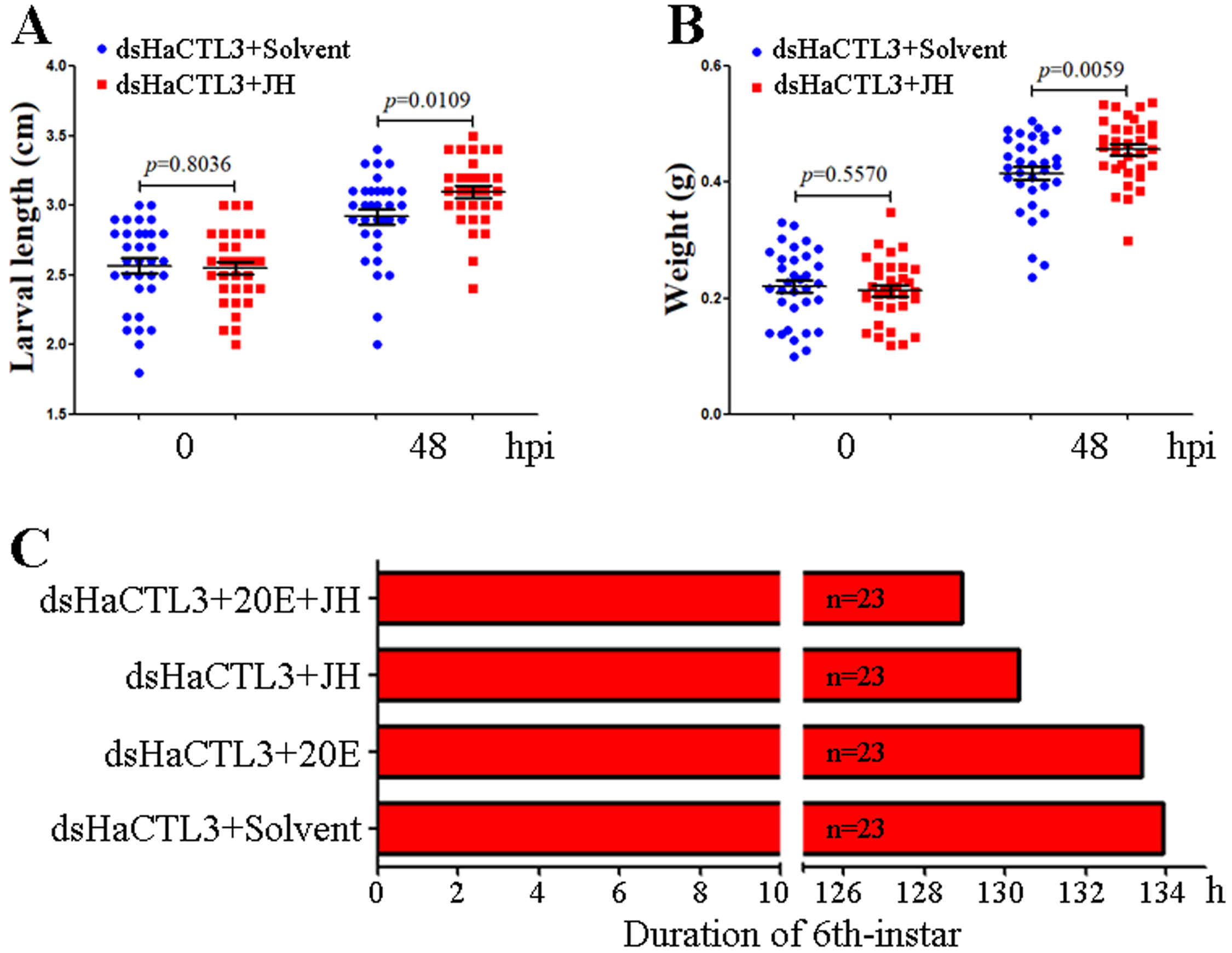

Supplement: S2 Fig — (A, B) JH treatment elevates body length (A) and weight (B) in HaCTL3-depleted larvae. Larval body length and weight were measured at 0 and 48 h post injection (hpi) of JH or DMSO (as solvent control). The p value was calculated by the Student′s t test for paired samples, and a p value of < 0.05 was considered statistically significant. (C) JH or 20E plus JH treatment accelerates pupation of HaCTL3-depleted larvae. The duration of sixth-instar was measured based on 23 20E-, JH-, 20E plus JH-, or solvent-injected larvae pretreated with dsHaCTL3. (TIF) [file ppat.1008901.s002.tif]

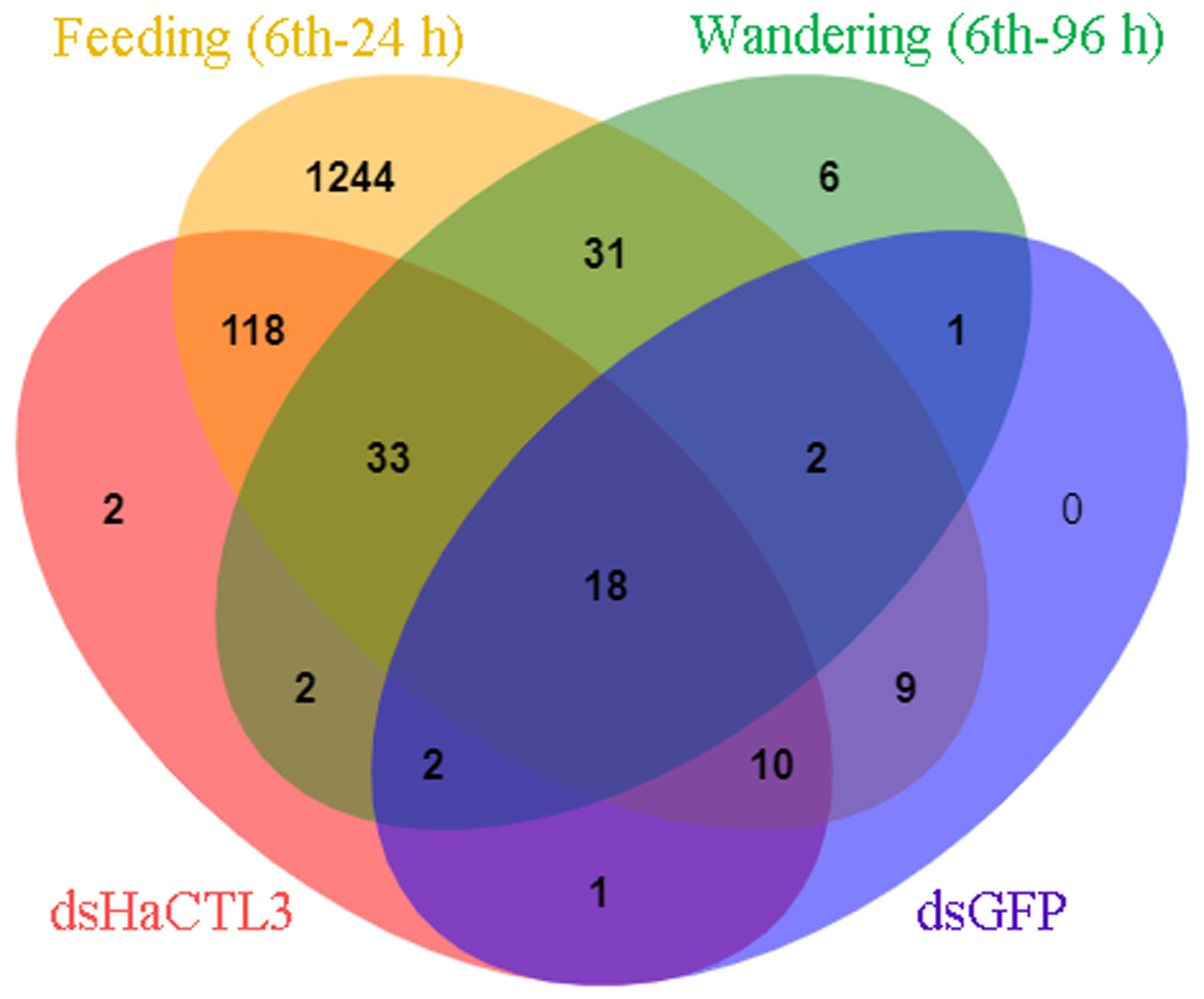

Supplement: S3 Fig — Hemolymph was collected from sixth-instar larvae at 24 h PE (6th-24 h; Feeding) and 96 h PE (6th-96 h; Wandering), as well as from larvae of dsHaCTL3- or dsGFP-injected (as control) groups. (TIF) [file ppat.1008901.s003.tif]

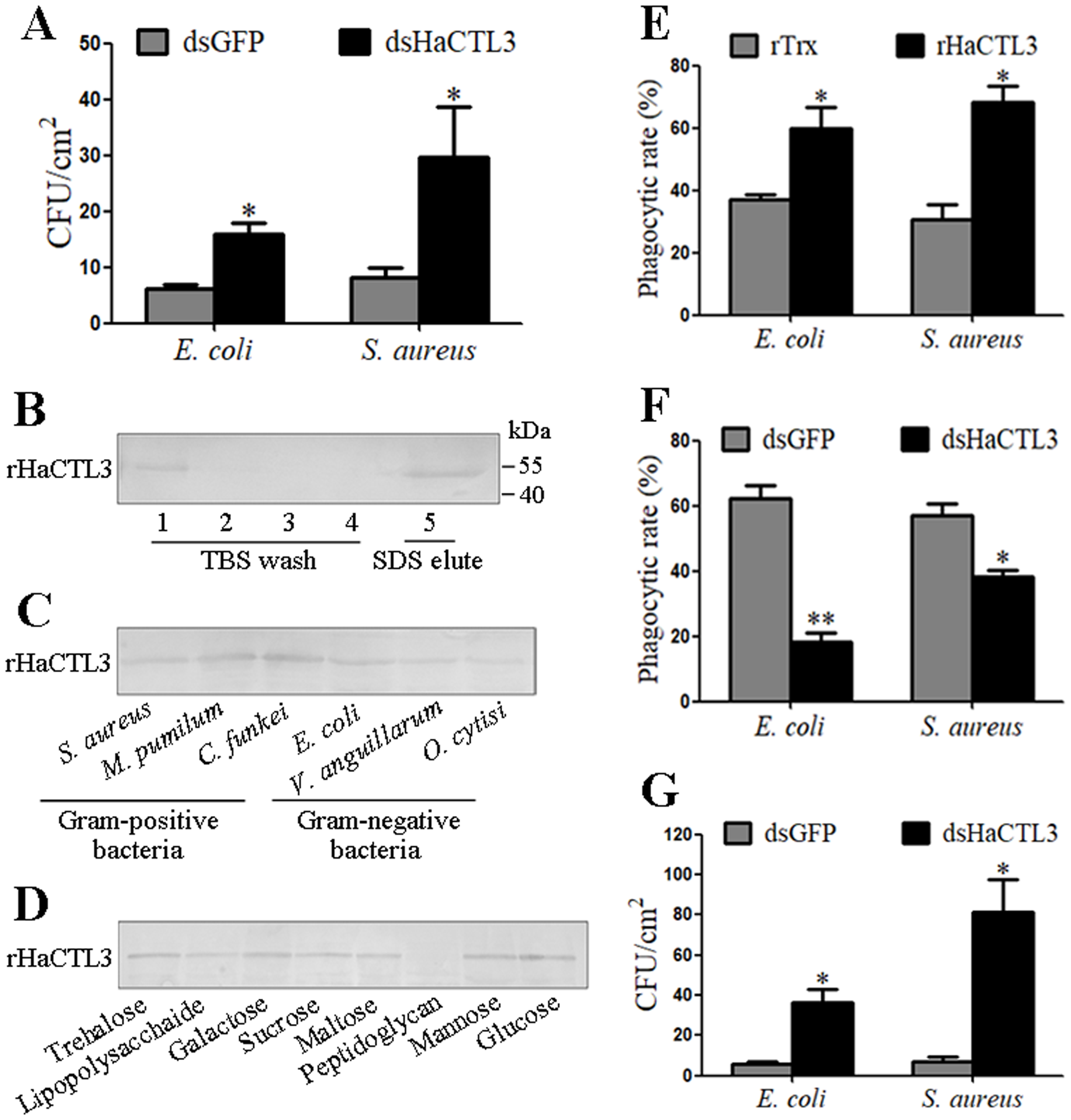

Supplement: S4 Fig — (A) Depletion of HaCTL3 suppresses bacterial clearance in the hemocoel of H. armigera larvae. Larvae pretreated with dsHaCTL3 or dsGFP were injected with E. coli or S. aureus. Hemolymph was collected at 1 h post-bacterial injection, and the number of CFUs was determined. (B) Binding of rHaCTL3 to S. aureus. S. aureus were incubated with rHaCTL3 for 30 min. After washing with TBS four times, S. aureus were pelleted and subjected to elution with 7% SDS. Lanes 1–4, TBS wash solution; lane 5, 7% SDS elution. (C) Binding of rHaCTL3 to various bacteria. Various bacteria were incubated with rHaCTL3, washed with TBS, and eluted with 7% SDS. (D) Inhibition analyses of the binding ability of rHaCTL3 to S. aureus by carbohydrates. Each carbohydrate was incubated with rHaCTL3 followed by addition of S. aureus. PGN exhibited a competitively inhibitory effect on the binding of rHaCTL3 to S. aureus. Samples were applied for detection by anti-His antibody. (E) rHaCTL3 promotes hemocytic phagocytosis of different bacteria. FITC-labeled E. coli or S. aureus were preincubated with rHaCTL3 or rTrx and then injected into the hemocoel of sixth-instar larvae at 24 h PE. (F) Depletion of HaCTL3 suppresses hemocytic phagocytosis. Larvae at 96 h post-dsHaCTL3 or dsGFP treatment were injected with FITC-labeled E. coli or S. aureus. (G) Depletion of HaCTL3 suppresses antibacterial activities in the plasma. Cell-free plasma was obtained from larvae pretreated with dsHaCTL3 or dsGFP. After incubation with E. coli or S. aureus for 1 h, the plasma-bacterial suspension was plated, and the number of CFUs was recorded. Columns represent the mean of three individual counts ± SEM. *0.01 < p < 0.05, **0.001 < p < 0.01 (Student’s t-test). (TIF) [file ppat.1008901.s004.tif]

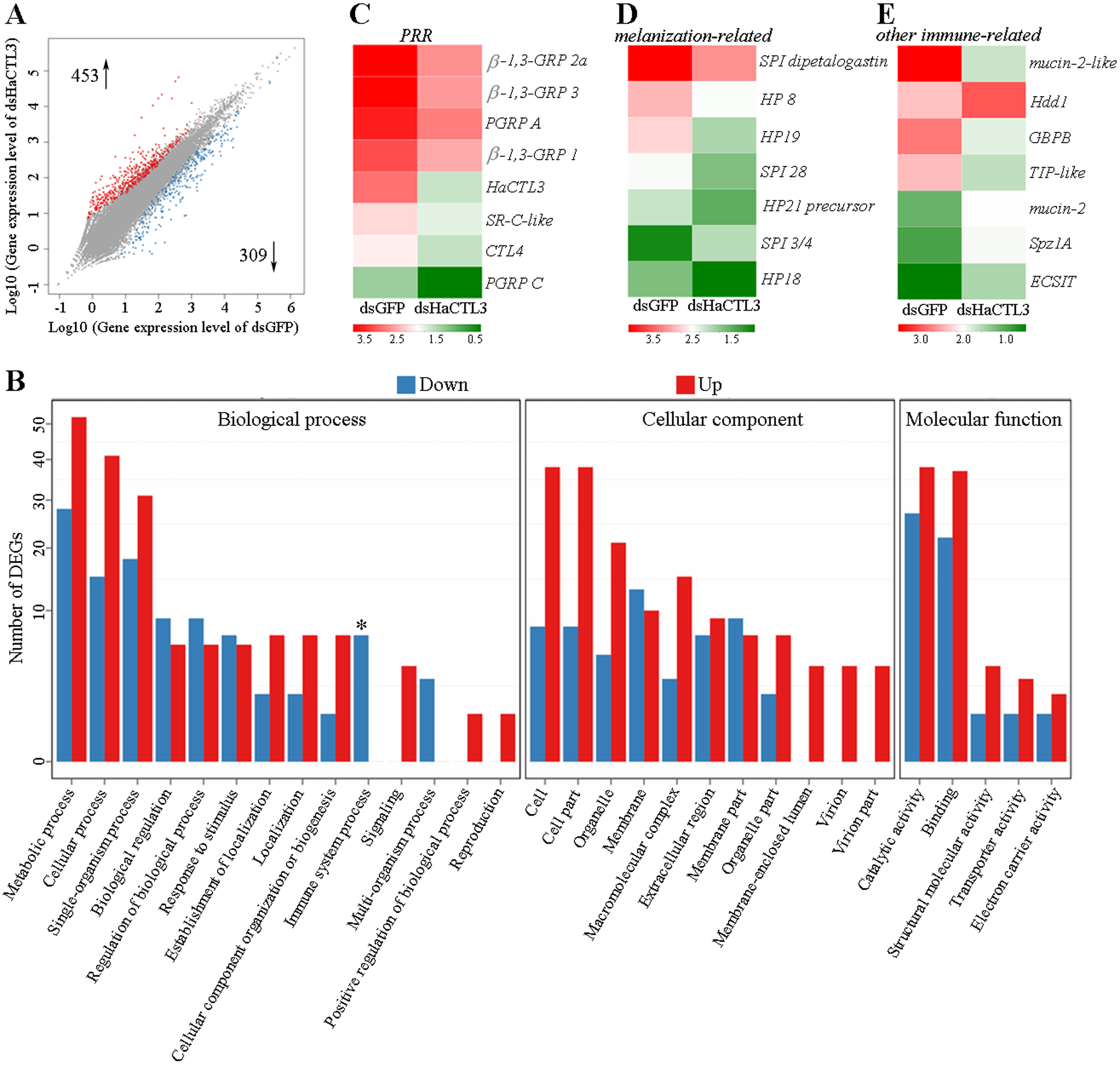

Supplement: S5 Fig — (A) Scatter plot represents DEGs in the HaCTL3-depleted fat body. DEGs were accepted with a cut-off of |log2FoldChange (dsHaCTL3/dsGFP)| ≥ 1 and Padj ≤ 0.05. (B) GO classification of DEGs in HaCTL3-depleted fat body. DEGs were assigned into three main GO terms, namely, biological process, cellular component, and molecular function, which were further subdivided into 30 subcategories. Asterisk represents that DEGs involved in immune system process were downregulated. (C-E) Heatmap showing that DEGs encoding PRRs (C), melanization-related proteins (D), and other immune-related proteins (E) varied in the HaCTL3-depleted fat body compared with that in the control sample, as characterized by RNA-seq analysis. (TIF) [file ppat.1008901.s005.tif]

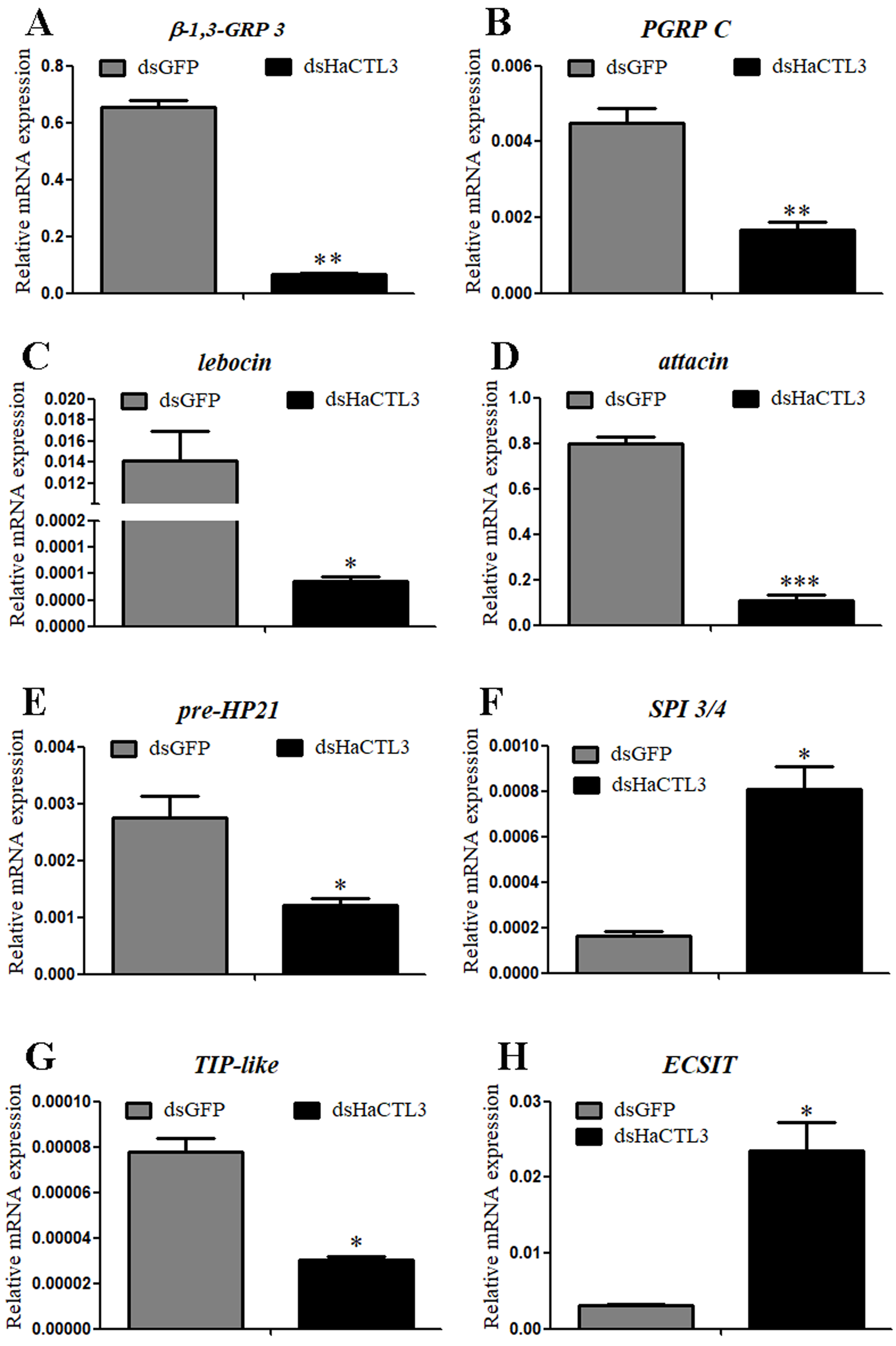

Supplement: S6 Fig — Randomly selected 2 PRRs (A, B), 2 AMPs (C, D), 2 melanization-related (E, F) and 2 other immune-related (G, H) DEGs were analyzed in response to HaCTL3 depletion. Error bars represent ±SEM. *0.01 < p < 0.05, **0.001 < p < 0.01, ***p < 0.001 (Student’s t-test). (TIF) [file ppat.1008901.s006.tif]

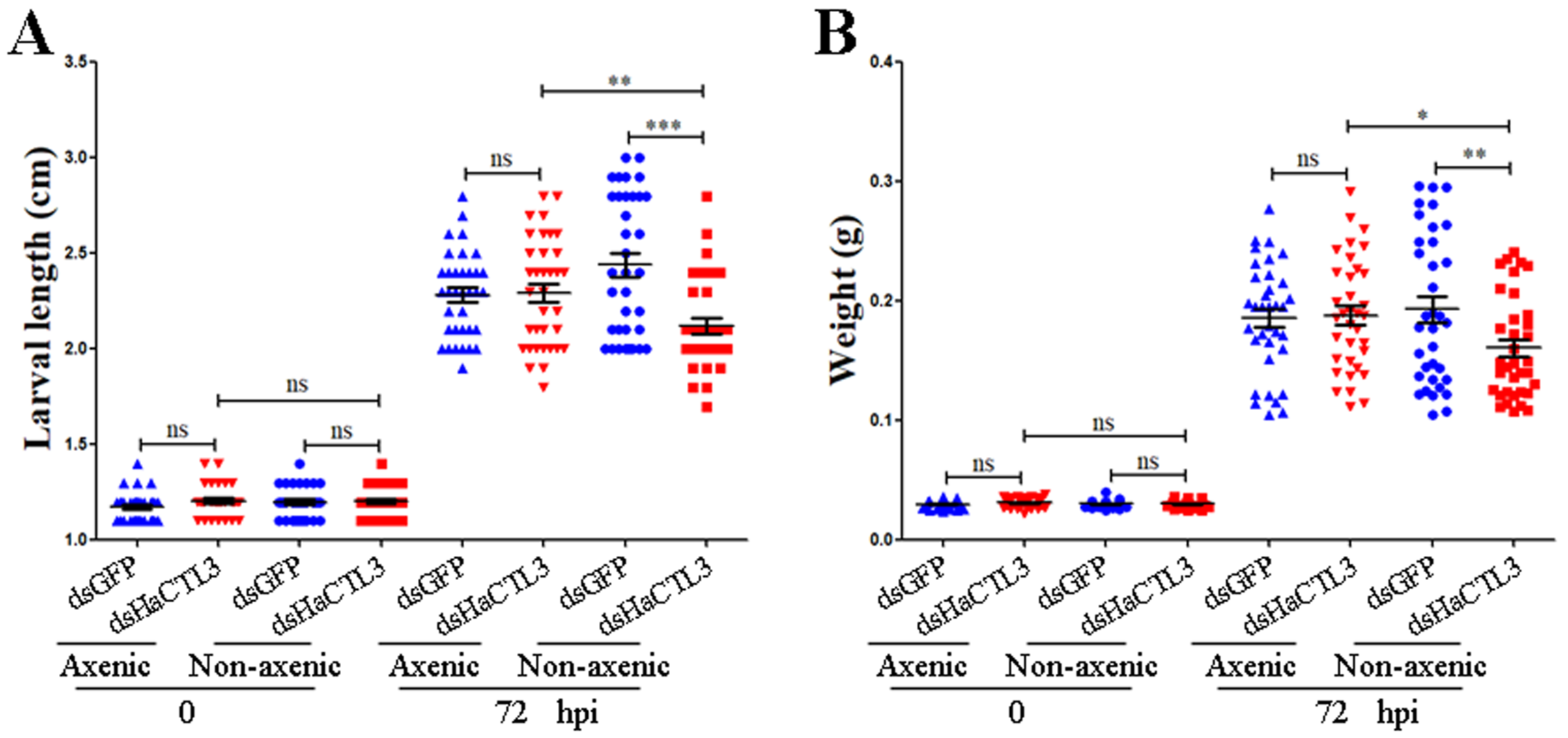

Supplement: S7 Fig — (A, B) Larval body length (A) and weight (B) were significantly reduced in HaCTL3-depleted nonaxenic larvae when compared with that in HaCTL3-depleted axenic larvae, or compared with that in nonaxenic larvae treated with dsGFP. Larval body length and weight were measured in axenic and nonaxenic larvae treated with dsHaCTL3 or dsGFP at 0 and 72 h post-dsRNA injection (hpi). *0.01 < p < 0.05, **0.001 < p < 0.01, ***p < 0.001 (Student’s t-test). (TIF) [file ppat.1008901.s007.tif]
